# Supplementary material for: Linkage to primary care after home-based blood pressure screening in rural KwaZulu-Natal, South Africa: a population-based cohort study
Source: BMJ Open. 2018 Dec 6;8(12):e023369. doi: 10.1136/bmjopen-2018-023369 (PMC6286496; doi:10.1136/bmjopen-2018-023369)
Supplement: Supplementary data [file bmjopen-2018-023369supp001.pdf]

Supplementary Table 1. Comparison of characteristics between those for whom a blood pressure measurement was taken in the 2010 survey, and those with no blood pressure measurements

	Eligible for survey[1]	BP measurements	No BP measurement[2]
	**N=26,184**	**N=11,694**	**N=14,490**
**Median (IQR) age (years)**	32 (20–50)	34 (21–53)	30 (20–47)
**Age group**			**P<0.001[3]**
<30	12,164 (46.5%)	5106 (43.7%)	7058 (48.7%)
35-44	5523 (21.1%)	2194 (18.8%)	3329 (23.0%)
45-59	4584 (17.5%)	2251 (19.2%)	2333 (16.1%)
60+	3913 (14.9%)	2143 (18.3%)	1770 (12.2%)
**Sex**			**P<0.001**
Male	9959 (38.0%)	3453 (29.5%)	6506 (44.9%)
Female	16,225 (62.0%)	8241 (70.5%)	7984 (55.1%)
**Marital status**			**P<0.001**
Single (never married)	8113 (31.2%)	3462 (29.8%)	4651 (32.4%)
Married/informal union	14,959 (57.6%)	6556 (56.3%)	8403 (58.6%)
Widow/sep/divorced	2910 (11.2%)	1618 (13.9%)	1292 (9.0 %)
Missing	*202*	*58*	*144*
**Education**			**P<0.001**
None	4558 (17.5%)	2389 (20.5%)	2169 (15.1%)
Less than complete secondary	13,466 (51.6%)	6244 (53.5%)	7222 (50.1%)
Complete secondary/above	8051 (30.9%)	3040 (26.0%)	5011 (34.8%)
Missing	*109*	*21*	*88*
**Employed**			**P<0.001**
Yes	5860 (22.7%)	1779 (15.3%)	4081 (28.6%)
No	19,997 (77.3%)	9828 (84.7%)	10,169 (71.4%)
Missing	*327*	*87*	*240*
**Residence**			**P<0.001**
Urban	1953 (7.5 %)	617 (5.3 %)	1336 (9.2 %)
Peri-urban	8084 (30.9%)	3604 (30.8%)	4480 (31.0%)
Rural	16,092 (61.6%)	7464 (63.9%)	8628 (59.7%)
Missing	*55*	*9*	*46*
**SES tertile**			**P<0.001**
Low	8566 (33.6%)	4193 (36.4%)	4373 (31.4%)
Middle	8330 (32.7%)	3818 (33.1%)	4512 (32.4%)
High	8569 (33.7%)	3522 (30.5%)	5047 (36.2%)
Missing	*719*	*161*	*558*

[1]Individuals who were on the eligibility list for the 2010 survey (aged ≥15 years as of Dec 2009 and resident in the DSS), were successfully contacted (92% of all on the list) and still eligible at the time of contact (75% of those contacted). [2]Includes 14,370 individuals who refused consent, and 120 individuals who consented but for whom blood pressure measurements were not available. [3]P-value from Chi-squared test comparing those with blood pressure measurements and those without.

Supplementary Table 2. Comparison of characteristics between those who participated in the general health survey in 2011 or 2012 and those who did not, among 1706 individuals with undiagnosed hypertension in 2010

	Participated in 2011/2012	Did not participate in 2011/2012	Participated in 2011–2016	No participation in later survey
	**N=1199 (70.3%)**	**N=507 (29.7%)**	**N=1421 (83.3%)**	**N=285 (16.7%)**
**Median (IQR) age (years)**	50 (38–66)	43 (29–58)	50 (37–65)	41 (28–57)
**Age group**		P<0.001[1]		P<0.001[2]
<30	193 (16.1%)	140 (27.6%)	248 (17.5%)	85 (29.8%)
35-44	242 (20.2%)	124 (24.5%)	284 (20.0%)	82 (28.8%)
45-59	360 (30.0%)	125 (24.7%)	430 (30.3%)	55 (19.3%)
60+	404 (33.7%)	118 (23.3%)	459 (32.3%)	63 (22.1%)
**Sex**		P<0.001		P<0.001
Male	308 (25.7%)	234 (46.2%)	394 (27.7%)	148 (51.9%)
Female	891 (74.3%)	273 (53.8%)	1027 (72.3%)	137 (48.1%)
**Marital status**		P<0.001		P<0.001
Single (never married)	250 (20.9%)	94 (18.7%)	287 (20.2%)	57 (20.2%)
Married/informal union	666 (55.5%)	342 (67.9%)	817 (57.5%)	191 (67.7%)
Widow/sep/divorced	283 (23.6%)	68 (13.5%)	317 (22.3%)	34 (12.1%)
**Education**		P<0.001		P<0.001
None	363 (30.3%)	119 (23.6%)	424 (29.8%)	58 (20.5%)
Less than complete secondary	581 (48.5%)	220 (43.6%)	678 (47.7%)	123 (43.5%)
Complete secondary/above	255 (21.3%)	166 (32.9%)	319 (22.4%)	102 (36.0%)
**Employed**		P<0.001		P<0.001
Yes	178 (14.8%)	119 (23.6%)	220 (15.5%)	77 (27.3%)
No	1021 (85.2%)	385 (76.4%)	1201 (84.5%)	205 (72.7%)
**Residence**		P=0.04		P=0.16
Urban	36 (3.0 %)	28 (5.5 %)	48 (3.4 %)	16 (5.6 %)
Peri-urban	398 (33.2%)	159 (31.4%)	470 (33.1%)	87 (30.6%)
Rural	765 (63.8%)	319 (63.0%)	903 (63.5%)	181 (63.7%)
**SES tertile**		P=0.24		P=0.05
Low	459 (38.7%)	189 (38.1%)	544 (38.7%)	104 (37.5%)
Middle	364 (30.7%)	171 (34.5%)	431 (30.7%)	104 (37.5%)
High	364 (30.7%)	136 (27.4%)	431 (30.7%)	69 (24.9%)
**BMI category**		P=0.008		P<0.001
Underweight	50 (5.7 %)	25 (7.2 %)	58 (5.6 %)	17 (9.0 %)
Normal weight	294 (33.6%)	146 (42.3%)	351 (34.0%)	89 (47.3%)
Overweight	229 (26.2%)	84 (24.3%)	269 (26.1%)	44 (23.4%)
Obese	301 (34.4%)	90 (26.1%)	353 (34.2%)	38 (20.2%)
**Distance to nearest clinic (km)**		P=0.22		P=0.50
0- <1.5	263 (21.9%)	121 (23.9%)	320 (22.5%)	64 (22.5%)
1.5-2.5	269 (22.4%)	127 (25.1%)	323 (22.7%)	73 (25.7%)
>2.5-3.9	310 (25.9%)	131 (25.9%)	365 (25.7%)	76 (26.8%)
>3.9	357 (29.8%)	127 (25.1%)	413 (29.1%)	71 (25.0%)

[1]P-value from Chi-squared test comparing individuals who participated in 2011-2012 (N=1199) with those who did not (N=507). [2]P-value from Chi-squared test comparing individuals who participated in 2011-2015 (N=1421) with those who did not (N=285).

Supplementary Table 3. Factors[1] associated with linkage to hypertension care within 2 years after home-based diagnosis of hypertension[2] in 2010, among individuals who were previously undiagnosed and participated in 2011 or 2012 (N=1199), using inverse probability weighting to account for non-participation in the blood pressure screen

	Linked to care/N (%)	Crude OR (95% CI)[3]	Age- & sex-adjusted OR (95% CI)[3]	Adjusted OR (95% CI)[3,4]
**Sociodemographic factors**				
**Age group**		P<0.001	P<0.001	P<0.001
<30	10 / 193 (5.2 %)	1	1	1
35-44	40 / 242 (16.5%)	3.82 (1.85 -7.87)	3.20 (1.55 -6.62)	3.26 (1.54 -6.90)
45-59	126 / 360 (35.0%)	10.35 (5.28 -20.32)	8.64 (4.39 -16.99)	9.35 (4.73 -18.46)
60+	167 / 404 (41.3%)	13.75 (7.05 -26.81)	12.27 (6.29 -23.91)	13.48 (6.82 -26.61)
**Sex**		P<0.001	P<0.001	P<0.001
Male	47 / 308 (15.3%)	1	1	1
Female	296 / 891 (33.2%)	2.86 (2.03 -4.03)	2.57 (1.81 -3.66)	2.77 (1.91 -4.00)
**Marital status**		P<0.001	P=0.09	P=0.09
Single (never married)	55 / 250 (22.0%)	1	1	1
Married/informal union	178 / 666 (26.7%)	1.35 (0.95 -1.91)	1.35 (0.91 -1.99)	1.36 (0.92 -2.01)
Widow/sep/divorced	110 / 283 (38.9%)	2.40 (1.63 -3.54)	0.94 (0.61 -1.44)	0.95 (0.61 -1.45)
**Education**		P<0.001	P=0.86	P=0.89
None	132 / 363 (36.4%)	1	1	1
Less than complete secondary	160 / 581 (27.5%)	0.64 (0.48 -0.84)	1.09 (0.80 -1.49)	1.07 (0.78 -1.46)
Complete secondary/above	51 / 255 (20.0%)	0.42 (0.29 -0.61)	1.06 (0.69 -1.63)	1.10 (0.71 -1.70)
**Employed**		P<0.001	P=0.01	P=0.01
Yes	31 / 178 (17.4%)	1	1	1
No	312 / 1021 (30.6%)	2.21 (1.46 -3.34)	1.74 (1.13 -2.69)	1.76 (1.14 -2.72)
**SES tertile**		P=0.36	P=0.32	P=0.26
Low	125 / 459 (27.2%)	1	1	1
Middle	99 / 364 (27.2%)	1.01 (0.74 -1.38)	1.11 (0.79 -1.55)	1.12 (0.81 -1.57)
High	115 / 364 (31.6%)	1.23 (0.90 -1.66)	1.28 (0.93 -1.77)	1.31 (0.95 -1.82)
**Location factors**				
**Residence**		P=0.03	P=0.40	P=0.68
Urban	10 / 36 (27.8%)	1	1	1
Peri-urban	95 / 398 (23.9%)	0.91 (0.42 -1.96)	0.84 (0.37 -1.89)	0.73 (0.32 -1.67)
Rural	238 / 765 (31.1%)	1.32 (0.62 -2.79)	1.03 (0.47 -2.27)	0.69 (0.30 -1.59)
**Nearest clinic (km)** [5]				
0- <1.5	56 / 263 (21.3%)			
1.5-2.5	71 / 269 (26.4%)	P<0.001	P=0.001	P=0.002
>2.5-3.9	93 / 310 (30.0%)	1.15 (1.08 -1.23) [6]	1.13 (1.05 -1.21) [6]	1.12 (1.04 -1.20) [6]
>3.9	123 / 357 (34.5%)			
**Clinical factors**				
**BMI category**		P<0.001	P=0.06	P=0.14
<25 kg/m^2	70 / 344 (20.3%)	1	1	1
25 – <30 kg/m^2	62 / 229 (27.1%)	1.56 (1.05 -2.33)	1.13 (0.73 -1.74)	1.19 (0.77 -1.84)
≥30 kg/m^2	110 / 301 (36.5%)	2.42 (1.69 -3.45)	1.57 (1.05 -2.34)	1.51 (1.00 -2.28)
**Hypertension stage**[7]		P<0.001	P<0.001	P<0.001
Stage I	142 / 730 (19.5%)	1	1	1
Stage II	134 / 342 (39.2%)	2.72 (2.04 -3.63)	2.20 (1.62 -2.99)	2.15 (1.58 -2.93)
Hypertension urgency	67 / 127 (52.8%)	4.79 (3.22 -7.13)	3.14 (2.06 -4.78)	3.10 (2.04 -4.71)
**Self-report of diabetes**[8]		P=0.28	P=0.50	P=0.56
No	339 / 1191 (28.5%)	1	1	1
Yes	4 / 8 (50.0%)	2.16 (0.53 -8.83)	1.56 (0.43 -5.65)	1.48 (0.40 -5.52)

**Self-report of TB[8]**		P=0.81	P=0.77	P=0.48
No	332 / 1156 (28.7%)	1	1	1
Yes	11 / 43 (25.6%)	0.92 (0.45 -1.86)	1.12 (0.51 -2.49)	1.34 (0.59 -3.04)

[1]All characteristics are based on 2010 survey. [2]Hypertension defined as systolic BP ≥ 140mmHg or diastolic BP ≥90 mmHg, in an average of 2 readings. [3]Weighted for non-response; weights calculated as the inverse probability of survey participation, in strata defined by age group, sex, education level and place of residence.
[4]Sociodemographic factors adjusted for age group, sex, marital status and employment. Location factors adjusted for age group, sex, marital status, employment, and distance from nearest clinic as continuous covariate. Clinical factors adjusted for age group, sex, marital status, employment, distance from nearest clinic, and hypertension stage. [5]Quartiles based on distribution in all individuals who were eligible for 2010 survey. Fit as continuous covariate; n (%) linked in each distance quartile shown for information only. [6]OR for linear trend in linkage with each 1 km increase in distance. [7]Stage I: Systolic BP 140–159 or diastolic BP 90–99; Stage II: Systolic BP 160–179 or diastolic BP 100–119; Hypertension urgency: Systolic BP ≥180 or diastolic BP ≥120. [8]Reports being diagnosed in the past 12m or currently on treatment.

Supplementary Table 4. Factors[1] associated with linkage to hypertension care within 5 years (2011 to 2015) after home-based diagnosis of hypertension[2] in 2010, among individuals who were previously undiagnosed and participated in a subsequent survey (N=1421) using inverse probability weighting to account for non-participation in the blood pressure screen

	Linked to care/N (%)	Crude OR (95% CI)[3]	Age- & sex-adjusted OR (95% CI)[3]	Adjusted OR (95% CI)[3,4]
**Sociodemographic factors**				
**Age group**		P<0.001	P<0.001	P<0.001
<30	25 / 248 (10.1%)	1	1	1
35-44	75 / 284 (26.4%)	3.22 (1.97 -5.29)	2.69 (1.64 -4.43)	2.93 (1.77 -4.87)
45-59	211 / 430 (49.1%)	8.67 (5.48 -13.70)	7.50 (4.73 -11.89)	8.05 (5.05 -12.84)
60+	265 / 459 (57.7%)	12.37 (7.84 -19.53)	11.65 (7.35 -18.47)	11.49 (7.25 -18.22)
**Sex**		P<0.001	P<0.001	P<0.001
Male	92 / 394 (23.4%)	1	1	1
Female	484 / 1027 (47.1%)	3.06 (2.35 -3.99)	2.98 (2.25 -3.94)	2.85 (2.15 -3.79)
**Marital status**		P<0.001	P=0.66	P=0.64
Single (never married)	98 / 287 (34.1%)	1	1	1
Married/informal union	298 / 817 (36.5%)	1.16 (0.88 -1.55)	1.04 (0.76 -1.44)	1.07 (0.77 -1.48)
Widow/sep/divorced	180 / 317 (56.8%)	2.78 (1.99 -3.87)	0.90 (0.61 -1.32)	0.92 (0.62 -1.35)
**Education**		P<0.001	P=0.55	P=0.66
None	217 / 424 (51.2%)	1	1	1
Less than complete secondary	269 / 678 (39.7%)	0.60 (0.47 -0.76)	1.13 (0.85 -1.50)	1.13 (0.86 -1.50)
Complete secondary/above	90 / 319 (28.2%)	0.36 (0.26 -0.49)	0.98 (0.68 -1.41)	1.05 (0.73 -1.53)
**Employed**		P<0.001	P=0.007	P=0.007
Yes	61 / 220 (27.7%)	1	1	1
No	515 / 1201 (42.9%)	2.04 (1.48 -2.81)	1.62 (1.14 -2.29)	1.62 (1.14 -2.29)
**SES tertile**		P=0.49	P=0.71	P=0.58
Low	224 / 544 (41.2%)	1	1	1
Middle	163 / 431 (37.8%)	0.89 (0.68 -1.15)	0.95 (0.71 -1.27)	0.97 (0.73 -1.30)
High	183 / 431 (42.5%)	1.04 (0.80 -1.35)	1.08 (0.81 -1.43)	1.13 (0.85 -1.50)
**Location factors**				
**Residence**		P=0.07	P=0.89	P=0.61
Urban	18 / 48 (37.5%)	1	1	1
Peri-urban	173 / 470 (36.8%)	1.03 (0.56 -1.92)	0.95 (0.50 -1.79)	0.84 (0.44 -1.62)
Rural	385 / 903 (42.6%)	1.34 (0.73 -2.46)	1.01 (0.54 -1.88)	0.75 (0.39 -1.46)
**Nearest clinic (km)** [5]				
0- <1.5	111 / 320 (34.7%)			
1.5-2.5	128 / 323 (39.6%)	P=0.001	P=0.03	P=0.05
>2.5-3.9	147 / 365 (40.3%)	1.10 (1.04 -1.17)[6]	1.08 (1.01 -1.15)[6]	1.07 (1.00 -1.14)[6]
>3.9	190 / 413 (46.0%)			
**BMI category**		P<0.001	P=0.004	P=0.008
<25 kg/m^2	133 / 409 (32.5%)	1	1	1
25 – <30 kg/m^2	99 / 269 (36.8%)	1.28 (0.92 -1.77)	0.88 (0.61 -1.26)	0.90 (0.62 -1.31)
≥30 kg/m^2	186 / 353 (52.7%)	2.44 (1.81 -3.29)	1.55 (1.10 -2.18)	1.53 (1.08 -2.17)
**Hypertension stage**[7]		P<0.001	P<0.001	P<0.001
Stage I	264 / 863 (30.6%)	1	1	1
Stage II	215 / 415 (51.8%)	2.49 (1.95 -3.17)	2.01 (1.54 -2.62)	2.12 (1.55 -2.89)
Hypertension urgency	97 / 143 (67.8%)	5.02 (3.42 -7.37)	3.18 (2.10 -4.82)	3.29 (2.01 -5.39)
**Self-report of diabetes**[8]		P=0.05	P=0.08	P=0.32
No	568 / 1410 (40.3%)	1	1	1
Yes	8 / 11 (72.7%)	3.84 (0.98 -15.03)	3.09 (0.88 -10.81)	5.18 (0.21 -128.15)

**Self-report of TB[8]**		P=0.73	P=0.86	P=0.30
No	560 / 1376 (40.7%)	1	1	1
Yes	16 / 45 (35.6%)	0.89 (0.48 -1.68)	1.07 (0.49 -2.33)	1.57 (0.67 -3.71)

[1]All characteristics are based on 2010 survey. [2]Hypertension defined as systolic BP ≥ 140mmHg or diastolic BP ≥90 mmHg, in an average of 2 readings. [3]Weighted for non-response; weights calculated as the inverse probability of survey participation, in strata defined by age group, sex, education level and place of residence.
[4]Sociodemographic factors adjusted for age group, sex, and employment. Location factors adjusted for age group, sex, employment, and distance from nearest clinic as continuous covariate. Clinical factors adjusted for age group, sex, employment, distance from nearest clinic, hypertension stage, and BMI. [5]Quartiles based on distribution in all individuals who were eligible for 2010 survey. Fit as continuous covariate; n (%) linked in each distance quartile shown for information only. [6]OR for linear trend in linkage with each 1 km increase in distance. [7]Stage I: Systolic BP 140–159 or diastolic BP 90–99; Stage II: Systolic BP 160–179 or diastolic BP 100–119; Hypertension urgency: Systolic BP ≥180 or diastolic BP ≥120. [8]Reports being diagnosed in the past 12m or currently on treatment.

Supplementary Table 5. Factors[1] associated with linkage to hypertension care within 5 years (2011 to 2015) after home-based diagnosis of hypertension[2] in 2010, among individuals who were previously undiagnosed and participated in a subsequent survey (N=1421)

	Linked to care/N (%)	Crude OR (95% CI)[3]	Adjusted OR (95% CI)[3,4]	Adjusted OR (95% CI)[3,5]
**Sociodemographic factors**				
**Age group**		P<0.001	P<0.001	P<0.001
<30	25 / 248 (10.1%)	1	1	1
35-44	75 / 284 (26.4%)	3.22 (1.97 -5.29)	2.43 (1.47 -4.01)	2.61 (1.56 -4.34)
45-59	211 / 430 (49.1%)	8.67 (5.48 -13.70)	6.58 (4.13 -10.47)	6.96 (4.35 -11.14)
60+	265 / 459 (57.7%)	12.37 (7.84 -19.53)	10.23 (6.44 -16.25)	10.11 (6.37 -16.06)
**Sex**		P<0.001	P<0.001	P<0.001
Male	92 / 394 (23.4%)	1	1	1
Female	484 / 1027 (47.1%)	3.06 (2.35 -3.99)	2.54 (1.91 -3.39)	2.46 (1.84 -3.30)
**Times participated**				
Once	61 / 321 (19.0%)			
Twice	119 / 335 (35.5%)	P<0.001	P<0.001	P<0.001
3 times	160 / 327 (48.9%)	1.57 (1.44 -1.71)[6]	1.40 (1.27 -1.53)[6]	1.39 (1.26 -1.52)[6]
4 times	147 / 287 (51.2%)			
5 times	89 / 151 (58.9%)			
**Marital status**		P<0.001	P=0.60	P=0.55
Single (never married)	98 / 287 (34.1%)	1	1	1
Married/informal union	298 / 817 (36.5%)	1.16 (0.88 -1.55)	1.14 (0.82 -1.60)	1.16 (0.83 -1.63)
Widow/sep/divorced	180 / 317 (56.8%)	2.78 (1.99 -3.87)	1.00 (0.67 -1.48)	1.01 (0.68 -1.50)
**Education**		P<0.001	P=0.74	P=0.67
None	217 / 424 (51.2%)	1	1	1
Less than complete secondary	269 / 678 (39.7%)	0.60 (0.47 -0.76)	1.12 (0.84 -1.49)	1.12 (0.84 -1.50)
Complete secondary/above	90 / 319 (28.2%)	0.36 (0.26 -0.49)	1.09 (0.75 -1.59)	1.16 (0.79 -1.69)
**Employed**		P<0.001	P=0.03	P=0.03
Yes	61 / 220 (27.7%)	1	1	1
No	515 / 1201 (42.9%)	2.04 (1.48 -2.81)	1.48 (1.04 -2.10)	1.48 (1.04 -2.10)
**SES tertile**		P=0.49	P=0.68	P=0.56
Low	224 / 544 (41.2%)	1	1	1
Middle	163 / 431 (37.8%)	0.89 (0.68 -1.15)	0.98 (0.73 -1.32)	1.00 (0.74 -1.35)
High	183 / 431 (42.5%)	1.04 (0.80 -1.35)	1.11 (0.83 -1.48)	1.15 (0.86 -1.54)
**Location factors**				
**Residence**		P=0.07	P=0.63	P=0.61
Urban	18 / 48 (37.5%)	1	1	1
Peri-urban	173 / 470 (36.8%)	1.03 (0.56 -1.92)	0.82 (0.43 -1.57)	0.76 (0.40 -1.48)
Rural	385 / 903 (42.6%)	1.34 (0.73 -2.46)	0.93 (0.49 -1.74)	0.85 (0.45 -1.61)
**Nearest clinic (km)[7]**				
0- <1.5	111 / 320 (34.7%)			
1.5-2.5	128 / 323 (39.6%)	P=0.001	P=0.06	P=0.10
>2.5-3.9	147 / 365 (40.3%)	1.10 (1.04 -1.17)[8]	1.07 (1.00 -1.14)[8]	1.06 (0.99 -1.13)[8]
>3.9	190 / 413 (46.0%)			
**BMI category**		P<0.001	P=0.003	P=0.007
<25 kg/m^2	133 / 409 (32.5%)	1	1	1
25 – <30 kg/m^2	99 / 269 (36.8%)	1.28 (0.92 -1.77)	0.88 (0.61 -1.28)	0.89 (0.61 -1.30)
≥30 kg/m^2	186 / 353 (52.7%)	2.44 (1.81 -3.29)	1.58 (1.11 -2.25)	1.54 (1.07 -2.22)
**Hypertension stage[9]**		P<0.001	P<0.001	P<0.001
Stage I	264 / 863 (30.6%)	1	1	1
Stage II	215 / 415 (51.8%)	2.49 (1.95 -3.17)	2.19 (1.67 -2.88)	2.36 (1.71 -3.26)
Hypertension urgency	97 / 143 (67.8%)	5.02 (3.42 -7.37)	3.44 (2.25 -5.25)	3.74 (2.25 -6.20)

**Self-report of diabetes[10]**		P=0.05	P=0.03	P=0.28
No	568 / 1410 (40.3%)	1	1	1
Yes	8 / 11 (72.7%)	3.84 (0.98 -15.03)	3.67 (1.14 -11.80)	5.64 (0.25 -126.50)
**Self-report of TB[10]**		P=0.73	P=0.91	P=0.56
No	560 / 1376 (40.7%)	1	1	1
Yes	16 / 45 (35.6%)	0.89 (0.48 -1.68)	0.96 (0.43 -2.13)	1.31 (0.52 -3.27)

[1]All characteristics are based on 2010 survey. [2]Hypertension defined as systolic BP ≥ 140mmHg or diastolic BP ≥90 mmHg, in an average of 2 readings. [3]Weighted for non-response; weights calculated as the inverse probability of survey participation, in strata defined by age group, sex, education level and place of residence. [4]Adjusted for age, sex and number of times participated in subsequent surveys as a continuous covariate. [5]Sociodemographic factors adjusted for age group, sex, number of times in subsequent surveys, and employment. Location factors adjusted for age group, sex, number of times in subsequent surveys, and employment. Clinical factors adjusted for age group, sex, number of times in subsequent surveys, employment, hypertension stage, and BMI. [6]OR for linear trend in reported linkage with each unit increase in survey participation. [7]Quartiles based on distribution in all individuals who were eligible for 2010 survey. Fit as continuous covariate; n (%) linked in each distance quartile shown for information only. [8]OR for linear trend in linkage with each 1 km increase in distance. [9]Stage I: Systolic BP 140–159 or diastolic BP 90–99; Stage II: Systolic BP 160–179 or diastolic BP 100–119; Hypertension urgency: Systolic BP ≥180 or diastolic BP ≥120. [10]Reports being diagnosed in the past 12m or currently on treatment.
