# Supplementary material for: Linkage to primary care after home-based blood pressure screening in rural KwaZulu-Natal, South Africa: a population-based cohort study
Source: BMJ Open. 2018 Dec 6;8(12):e023369. doi: 10.1136/bmjopen-2018-023369 (PMC6286496; doi:10.1136/bmjopen-2018-023369)
Supplement: Author's manuscript [file bmjopen-2018-023369.draft_revisions.pdf]

BMJ Open is committed to open peer review. As part of this commitment we make the peer review history of every article we publish publicly available.

When an article is published we post the peer reviewers' comments and the authors' responses online. We also post the versions of the paper that were used during peer review. These are the versions that the peer review comments apply to.

The versions of the paper that follow are the versions that were submitted during the peer review process. They are not the versions of record or the final published versions. They should not be cited or distributed as the published version of this manuscript.

BMJ Open is an open access journal and the full, final, typeset and author-corrected version of record of the manuscript is available on our site with no access controls, subscription charges or pay-per-view fees (http://bmjopen.bmj.com).

If you have any questions on BMJ Open's open peer review process please email info.bmjopen@bmj.com

BMJ Open

Linkage to primary care after home-based blood pressure screening in rural Kwazulu-Natal: A population-based cohort study

Journal:	*BMJ Open*
Manuscript ID	bmjopen-2018-023369
Article Type:	Research
Date Submitted by the Author:	13-Apr-2018
Complete List of Authors:	Siedner, Mark; Massachusetts General Hospital, Medicine; Harvard Medical School,<br>Baisley, Kathy; London School of Hygiene and Tropical Medicine, Epidemiology and Population Health<br>Orne-Gliemann, Joanna; University of Bordeaux<br>Pillay, Deenan; Africa Health Research Institute; University of College London<br>Koole, Olivier; Africa Health Research Institute<br>Wong, Emily; Massachusetts General Hospital<br>Matthews, Philippa; Africa Health Research Institute<br>Tanser, Frank; School of Nursing and Public Health, University of KwaZulu-Natal ; Africa Health Research Institute<br>Herbst, Kobus; Africa Health Research Institute<br>Barnighausen, Till; Africa Health Research Institute<br>Bachmann, Max; UEA, Population Health and Primary Care
Keywords:	Hypertension < CARDIOLOGY, sub-Saharan Africa, Linkage to care, Population health

SCHOLARONE™
Manuscripts

Linkage to primary care after home-based blood pressure screening in rural Kwazulu-Natal: A population-based cohort study

Authors:

Mark J. Siedner*[1,2,3]
Kathy Baisley[3,4]
Joanna Orne-Gliemann[5]
Deenan Pillay[3,6]
Olivier Koole[3,4]
Emily Wong[1,2,3]
Philippa Matthews[3]
Frank Tanser[3]
Kobus Herbst[3]
Till Barnighausen[3,6,7,8]
Max Bachmann[9]

*Corresponding Author for Inquiries:
Mark J. Siedner
Massachusetts General Hospital Global Health
125 Nashua Street
Boston, MA 02114 USA
T: +1-617-726-4686; F: +1-617-724-1637
msiedner@mgh.harvard.edu

Affiliations:
[1]Massachusetts General Hospital, Boston, USA
[2]Harvard Medical School, Boston, USA
[3]Africa Health Research Institute, KwaZulu-Natal, SA
[4]London School of Tropical Medicine and Hygiene, London, UK
[5]University of Bordeaux, ISPED, INSERM, Bordeaux Population Health Research Center, UMR 1219, Bordeaux, France
[6]University College London, London, UK
[7]University of Heidelberg, Germany
[8]Harvard School of Public Health, Boston, USA
[9]University of East Anglia, Norwich, UK

Funding:
The Africa Health Research Institute receives funding from the United Kingdom Wellcome Trust grant 082384/Z/07/Z. MJS receives funding from the National Institutes of Health (K23 MH099916, P30 30AI060354)

Conflicts of Interest: All authors report no conflicts of interest

For peer review only

ABSTRACT

Objectives: The expanding burden of non-communicable diseases (NCDs) globally will require novel public health strategies. Community-based screening has been promoted to augment efficiency of diagnostic services, but few data are available on the downstream impact of such programs. We sought to assess the impact of a home-based blood pressure screening program on linkage to hypertension care in rural South Africa.

Setting: We conducted home-based blood pressure screening in a population cohort in rural KwaZulu-Natal, using the WHO STEPS protocol.

Participants: Individuals meeting criteria for raised blood pressure (>140 systolic or >90 diastolic averaged over two readings) were referred to local health clinics, and included in this analysis. We defined linkage to care based on self-report of presentation to clinic for hypertension during the next two years of cohort observation. We estimated the population proportion of successful linkage to care with inverse probability sampling weights, and fit multivariable logistic regression models to identify predictors of linkage following a positive hypertension screen.

Results: Of 11,694 individuals screened, 15% (n=1,706) were newly diagnosed with elevated pressure. 26.9% (95%CI 24.5-29.4%) of those sought hypertension care in the following two years, and 38.1% (95%CI 35.6-40.7%) did so within five years. Women (aOR 2.41, 95%CI 1.68–3.45), those of older age (aOR 11.49, 95%CI 5.87–22.46, for 45-59 years versus <30), and those unemployed (aOR 1.71, 95%CI 1.10–2.65) were more likely to have linked to care.

Conclusions: Linkage to care after home-based identification of elevated blood pressure was rare in rural South Africa, particularly among younger individuals, men, and the employed. Improved understanding of barriers and facilitators to NCD care is needed to improve the effectiveness of blood pressure screening in the region.

Key Words:
Non-communicable diseases, community health, hypertension, South Africa, linkage to care

Strengths and Limitations of this Study

- Applies a longitudinal population cohort study design with a large sample size to assess linkage to hypertension care after a home-based screening for elevated blood pressure
- Assesses an under-studied population in rural sub-Saharan Africa who are known to have high prevalence of hypertension and low rates of engagement in hypertension care
- Identifies low rates of linkage to care after home-based blood pressure screening in this population, and key factors associated with poor linkage including male sex, younger age, and being employed
- Limitations include low rates of participation in the home-based screening program and incomplete follow-up, as well as self-reported linkage to care as an outcome definition

For peer review only - http://bmjopen.bmj.com/site/about/guidelines.xhtml

What is already known on this subject?

Hypertension is the risk factor responsible for the greatest number of deaths globally. Yet, awareness and treatment of the disease remains low, particularly in resource limited settings. Home and community-based hypertension screenings have gained major traction as a means to improve penetration of disease diagnosis and prevention. Yet, few studies have assessed their downstream effects on linkage to hypertension care.

What this study adds?

We found that less than 3 in 10 people newly notified of elevated blood pressure during a home-based screening in rural KwaZulu-Natal presented to a clinic for hypertension care within the next two years. Linkage rates were particularly low in younger people, men and the employed. Consequently, community and home-based blood pressure screening in similar settings will likely require additional features to result in their desired effect of improving access to effective hypertensive care.

For peer review only

BACKGROUND

Over two in three deaths worldwide are attributed to non-communicable diseases (NCDs).[1] Although precise measurement of cause-specific mortality in much of the developing world remains a challenge, some estimates suggest that the majority of NCD deaths now occur in low and middle-income countries.[2] In South Africa, for example, the World Health Organization estimates that half of deaths are due to NCDs, and approximately 25% of the population will suffer a premature death due to them.[3]

Consequently, responding to the NCD epidemic in low and middle-income countries is both a major challenge and stated priority of the public health community.[4] NCD morbidity and mortality can be substantially reduced through effective primary and secondary prevention measures targeting risk factors such as smoking, high blood pressure, diabetes, diet and physical activity.[5] Hypertension, which can be controlled through cost-effective lifestyle and pharmacotherapy interventions, is estimated to account for over 50% of the population attributable fraction of stroke in the African region.[6] Yet, in South Africa, national population surveys have estimated that over one quarter of South Africans adults have raised blood pressure, but only approximately one in three of them has received treatment.[7]

The South African Department of Health has outlined strategic NCD goals, which highlight the role for prevention of NCDs and the importance of a community-based focus.[8] One specific strategy includes integrating HIV and NCD screening programs and broadening access to diagnostic and treatment in the community and rural areas. Community-based NCD screening through health fairs and use of community health workers has gained traction recently as a means to efficiently screen large populations of individuals for multiple co-morbidities.[9 10] Whether such endeavors lead to successful linking of individuals to appropriate NCD care is not well established, and is an important question for the field.

In 2010, we conducted a home-based assessment of blood pressure in approximately 12,000 people in a demographic health surveillance (DHS) site in KwaZulu Natal. We referred individuals with raised blood

For peer review only

pressure and not already receiving hypertension treatment to local government clinics for repeat measurement and ongoing hypertension care. We assessed linkage to care during future years of the home-based DHS survey. Our primary aims were to determine the probability of clinical engagement within two years after home-based screening and referral, and to identify predictors of failure to link to care. Our over-arching aim was to inform public health programmers on the feasibility of community-based blood pressure screening as an entry point into NCD care in this setting.

METHODS

Study design, setting, and participants

The African Health Research Institute (AHRI) (formerly the Africa Centre for Health and Population Studies) is a Wellcome Trust funded research institute in South Africa. Since 2000, they have conducted a population cohort study of all adults in a catchment area of 438 km^2 in rural uMkanyakude District, northern KwaZulu-Natal, covering a total population of approximately 100,000 individuals.[11] Households are surveyed 2–3 times per year, to collect information on birth, deaths and migration patterns for all household members, including non-residents. Since 2003, resident household members ≥15 years have been invited to participate in an annual home-based individual survey, which collects data on sociodemographics and general health information.

Blood pressure screening and referral methods

In 2010, all individuals who participated in the home-based survey were also offered a physical examination to determine weight, height and blood pressure, using the WHO STEPS protocol.[12] Blood pressure was measured using Omron automated blood pressure monitors (Omron Global, Kyoto Japan). Blood pressure was measured after 15 minutes of resting in a seated position. We collected three measurements, each five minutes apart, with the mean of the last two measurements used to identify those with elevated blood pressure. A positive hypertension screen was defined as a mean systolic blood pressure ≥140 mmHg and/or diastolic blood pressure ≥90 mmHg.[13] Individuals with a positive screen and

For peer review only - http://bmjopen.bmj.com/site/about/guidelines.xhtml

not already receiving treatment were referred to their preferred local public health clinic with their screening results.

Outcome assessment

For our primary outcome of interest, we defined successful linkage to care for hypertension as self-reported linkage within two years of a positive home-based hypertension screen. To assess this outcome, we used data from the two subsequent, annual home-based health surveys in 2011 and 2012. In each annual health survey, respondents were asked if: 1) they have been diagnosed with hypertension in the past 12 months; 2) if they have ever received hypertension treatment; and 3) if they are currently being treated for hypertension. We defined successful linkage to care by a positive response to any of these three questions in either 2011 or 2012. As secondary outcomes of interest, we also examined 1) linkage to hypertension care within five years (as opposed to two), defined as a positive response to any of the same three questions in the annual health surveys during 2011-2015 and 2) confirmation of hypertension care seven years after the screening, as evidenced by clinical records from all public health clinics in the catchment area in 2017, the first year linked clinical data was linked to the population cohort database.

Statistical methods

We included in this analysis individuals who had a positive hypertension screen in the home-based 2010 survey, and who reported no previous diagnosis of hypertension or hypertension treatment. We first summarized sociodemographic characteristics of eligible adults who had blood pressure measurements in the 2010 survey. We then estimated population-level prevalence of linkage to hypertension care in the two years after the screening program, both overall and stratified by sex and age, with the use of inverse probability weights (IPWs) of the probability of participating in the hypertension screening. We used IPWs to make the results generalizable to the entire 2010 sample. To calculate the weights, we fit a logistic regression model with completion of blood pressure screening in 2010 as the outcome of interest and

For peer review only

included age strata, sex, education level and place of residence (urban, peri-urban, or rural) as predictors, based on information routinely collected in the household-level survey.

We then fit logistic regression models to estimate odds ratios (OR) and 95% confidence intervals (CI) for factors independently associated with linkage to hypertension care within two years of a positive home-based hypertension screen. Potential determinants of linkage were examined at three levels: sociodemographic factors (age, sex, educational attainment, employment status and socioeconomic status); geographical factors (distance from clinic, urban versus rural residency); and clinical factors (body mass index, elevated blood pressure severity [defined using hypertension stages as a) stage I: systolic 140-160 and diastolic 80-100; b) stage II: systolic 160-<180 or diastolic 100-<120; or c) hypertensive urgency: systolic >180 or diastolic >120], self-report of diabetes, self-report of tuberculosis)[14]. Sociodemographic and clinical factors whose age- and sex-adjusted association with linkage was significant at p<0.10 were included in a final adjusted multivariable model. Distance from the nearest clinic was analyzed as a continuous covariate. In order to allow for non-linear relationships between distance and linkage to care, we used fractional polynomial functions.[15]

We tested the robustness of our findings using several sensitivity analyses. First, we changed our outcome from self-reported linkage to care in 2011 or 2012 to 1) self-reported linkage to care at any time between 2011-2015, and 2) confirmation of a clinic appointment for hypertension in 2017 among those who remained a resident in the catchment area. Next, we compared characteristics of eligible individuals who did and did not complete blood pressure screening in 2010. Next, we compared characteristics of those who participated in a subsequent health survey and those who did not, either because of refusal, out-migration, or death. Finally, we conducted sensitivity analyses in which we: 1) used IPWs of screening in the models; and 2) added a covariate to indicate the number of individual health surveys participated in during 2011-2015. Data were entered and verified in an SQL database, and were analyzed using Stata 14 (StataCorp, College Station, TX).

For peer review only

Human Studies Considerations

Ethical approval for the demographic surveillance study and analyses of these data was granted by the Biomedical Research Ethics Committee of the University of KwaZulu-Natal, South Africa. Separate informed consent was given for the demographics survey, the blood pressure screening, and the clinic records abstraction.

Patient and Public Involvement

Patients were not involved in the design of this study. This analysis was designed by study investigators at the Africa Health Research Institute intent on leveraging prior home-based screening protocols to inform and optimize future community-based research, and particularly to improve the public health impact of such activities. The results of this study were presented to the South African Department of Health Non-Communicable Diseases Unit and will be disseminated to the community during the monthly scheduled Africa Health Research Institute community road shows.

RESULTS

Survey participants

A total of 37,693 potentially eligible adults were in the sampling frame. Of these, approximately one quarter (8,589, 23%) were not available due to out-migration, death or inability to consent and another 2,920 (8%) could not be contacted (Figure 1). Of the remaining 26,184 individuals who were contacted and eligible for the home-based DHS survey in 2010, 11,814 (45%) consented to participate in the general health survey and 11,694 (45%) had valid blood pressure measurements. Women, older individuals, and those of lower socioeconomic position and education were more likely to participate in the survey (Supplementary Table 1).

For peer review only

The majority of participants with a blood pressure measurement were women (n=8,241, 71%, Table 1). Median age was 25 years (interquartile range [IQR] 18–47 years) for men and 38 years (IQR 23–55 years) for women. The majority of participants (n=7,464, 64%) resided in a rural setting, and less than one quarter (n=2,642, 23%) lived within 1.5 kilometers of the nearest clinic. Few participants (n=1,779, 15%) were currently employed.

Screening for hypertension

Approximately one quarter (n=3,074, 26.2%) of participants were found to have elevated pressure during the home-based blood pressure screening, of whom 1,368 (44.5%) reported having been previously diagnosed or currently on treatment. Of those who had been previously diagnosed or in hypertension care, 1,169 (85.5%) were currently on hypertension treatment. Participants who were not previously aware of their condition were significantly younger, and more likely to be men, married, employed, have a higher level of education, and be living in peri-urban areas than those who had been previously diagnosed or on treatment (Table 1). However, there was no evidence of a difference between the two groups in the distance from their nearest clinic.

Analytic Sample

A total of 1,199 individuals (70.3%) who were not previously aware of having elevated blood pressure participated in a second general health survey within two years of being screened (that is, in 2011 or 2012), and were included in the primary analysis of factors associated with linkage to hypertension care. Compared with the 507 individuals who did not participate in 2011 or 2012, those who participated in 2011 or 2012 were older (median (IQR) age = 50 (38–66) years, vs 43 (29–58) years), more likely to be women, unmarried, have lower levels of education, be unemployed, and have a higher BMI (Supplementary Table 2). There was no difference in participation rates by distance from the nearest clinic. When we expanded the observation period to include surveys from 2011-2015, a total of 1,421 (83.3%) participated in at least one home-based annual general health survey. Of the 285 (16.7%) individuals who

For peer review only

did not participate in any health survey after 2010, 81 out-migrated and 36 died before the 2011 survey (Figure 1). The remaining 168 were eligible for at least one subsequent survey but refused participation.

Linkage to subsequent hypertension care

Using IPWs to estimate population level linkage to care, we estimate a population level prevalence of linkage to care within two years of the blood pressure screen of 26.9% (95%CI=24.5-29.4%). Women were more likely than men to link to care, and older individuals were more likely than younger individuals (Figure 2), such that we estimate that 44.9% (95%CI=39.4-50.5%) of women ≥60 years presented to care for hypertension in the next two years, versus only 3.0% (95%CI=1.1-7.7%) of men under 45. When we extended our surveillance period out to 2015, we estimate that 38.1% (95%CI=35.6-40.7%) of individuals reported linking to hypertension care within 5 years. Finally, we found that only 16.6% (95%CI=14.6-18.9%) of individuals who remained a resident in 2016 and who screened positive for elevated blood pressure in 2010 completed a clinic appointment for hypertension at one of the public health clinics in the catchment area in 2016.

Factors associated with presentation to hypertension care within two years

We found strong evidence that women (OR=2.76, 95%CI=1.97 – 3.88, p<0.001) and those of older age (OR=12.89, 95%C 6.62 – 25.11, p<0.0001, comparing those 45-59 years versus those <30) were more likely to present to hypertension care within two years of home-based diagnosis (Table 2). In adjusted analysis, the association with age and sex remained statistically significant, and there was no evidence that the effect of age on linkage to care differed between men and women (p-value for interaction=0.20, Figure 2). There was evidence that those who were unemployed were more likely to link to care (adjusted (a)OR 2.09, 95%CI 1.39 – 3.14). There was an association between distance from clinic and linkage to hypertension care such that odds of presentation increased as distance to the clinic increased (aOR for linear trend in linkage with each 1 km increase in distance = 1.12, 95%CI=1.05–1.20, p<0.001). The results of the fractional polynomial models suggested that the linear model adequately described the relationship

For peer review only - http://bmjopen.bmj.com/site/about/guidelines.xhtml

between presentation to care and distance. After adjusting for sociodemographic and location factors, we also found strong evidence that individuals with the equivalent of Stage II hypertension (aOR=2.20, 95%CI=1.63 – 2.97), and those meeting criteria for hypertensive urgency (aOR=3.07, CI=2.01 – 4.67) had higher odds of linking to care than those with the equivalent of Stage I hypertension. We found similar correlates of presentation to hypertension care (age, sex, distance from clinic, and employment) in sensitivity analyses with weighted models, and in models with a covariate for the number of follow-up surveys completed during 2011-2015 (Supplementary Tables 3-5).

DISCUSSION

We found very low rates of presentation to care after home-based identification of elevated blood pressure in rural KwaZulu Natal. Overall, less than one third of individuals newly identified with elevated blood pressure reported being diagnosed with hypertension or receiving treatment for elevated blood pressure from a clinic within two years, and less than one in five had evidence of visiting a clinic for hypertension care during a 12-month period seven years after the screen. Linkage rates were particularly low for men and young people. Notably, those employed and those closest to clinics also had poorer rates of linkage. These results highlight the important need to consider the determinants of healthcare access for NCDs in rural South Africa, and multi-faceted approaches to improve linkage to care after community-based NCD screening programs.

Linkage with clinical programs after community- and home-based disease screening for chronic disease in sub-Saharan Africa have demonstrated mixed results. Most evidence has come from the HIV field, in which linkage after home-based testing in pilot studies has been highly successful,[16] although lower rates are reported in community settings.[9 17] Studies reporting clinic attendance after hypertension screening have generally shown low rates of linkage to care. For example, in a large (n=6,000) health fair-based screening program in Uganda, 41% of participants with a new positive screen for elevated blood pressure linked to care.[9] A smaller pilot study in Kenya yielded higher linkage rates (74%, n=120) after community group-

For peer review only

initiated blood pressure screening.[18] Interpreting these contrasting results must be done with attention to the selection criteria of each. Whereas our procedures were home-based, the larger study from Uganda included self-referring individuals who had attended a health fair, and the Kenyan study operated through a peer microfinance program, in which NCD screening services were paired with agribusiness advice within pre-organized community groups. In the prior report most similar to ours, a large program in Malawi (n=27,305) that provided clinical referrals after home-based testing reported a 59% linkage rate within two weeks of a diagnosis of hypertension, although 30% of participants were already on treatment at the time of referral.[19] Moreover, approximately 50% dropped out of care within 6 months of linkage.

Predictors of presentation to care in our study reinforce much of the literature on health care access and engagement among vulnerable populations in sub-Saharan Africa. Lower engagement by younger individuals and men are well-established phenomena; and a public health challenge for the region.[20 21] Although it did not reach statistical significance, we also found evidence that those with greater social support, as evidenced by having a cohabitating partner, tended to be more likely to present to care. An unexpected finding was that those who were unemployed and those further from clinic were more likely to link to care. This finding contrasts with much of the data from the region on how distance from health services impacts linkage to and retention in care.[22-24] We hypothesize that these results illustrate competing demands between obligations to work and to access healthcare. Notably, a similar phenomenon was found in the Malawi home-based NCD screening study, in which rural participants had more than twice the odds of linkage to NCD care than their urban counterparts, and the most common reason stated for failure to link to care in urban areas was being too busy to attend clinic; reported in 34% of those not linked to care.[19] Employment is higher in male than in female South African blacks [25], and so may also contribute to the gender difference in linkage.

Our data do offer multiple potential strategies to improve linkage to care after home-based NCD screening. For example, a notable distinction between many community-based HIV and NCD diagnostic programs is

For peer review only

the degree of counseling and referral services provided after diagnosis. Hypertension referral services in our program and many others in the region are often limited to distribution of results and referral forms. In contrast, decades of standardizing in-depth HIV counseling services, and additional facilitated linkage strategies have significantly improved rates of linkage after a new HIV diagnosis.[26] Pilot studies of enhanced referral after community based NCD diagnoses have also shown promise in vulnerable populations in the United States,[27 28] and warrant investigation on a larger scale elsewhere. Similarly, our finding and that of others that hypertension linkage was less common in those employed presents a potential opportunity to consider expanded clinic service hours and/or community based management to improve NCD care in the region. Endeavors, such as the Centralised Chronic Medication Dispensing and Distribution recently launched by the KwaZulu Natal Department of Health, seek to overcome such barriers by delivering medicines to peoples' homes and workplaces, or setting up community-based medicine pickup points. Evaluations of the efficacy and sustainability of such programs will be of high importance to the field.

Our study is strengthened by a large sample size and the use of a home-based testing paradigm. The primary limitation to our analysis is the relatively low response rate in the initial hypertension screen and participation in subsequent surveys. We accounted for this limitation by comparing characteristics between participants and non-participants, and by using inverse probability weighting techniques to make population level inferences. Our study is also limited by a use of self-report to detect linkage to clinical care over the first two years of observation, which likely results in incomplete estimation of outcomes. Use of clinical records to assess long-term care seven years after the initial screen might also under-estimate appropriate maintenance in care for individuals who normalized their blood pressure in the interim. Finally, our results should be considered in the context of the low-resource, rural sub-Saharan Africa study stetting, but are unlikely to generalize more broadly to urban or higher resource regions.

For peer review only

In summary, we found very low rates of linkage to care after a population-level, home-based hypertension screen in rural KwaZulu Natal. Strategies focused on increased demand generation, particularly for younger individuals and men, augmented referral and linkage programs, and efforts to enhance the convenience of service delivery, particularly to employed people, should be evaluated to improve NCD care access after community based testing in the region.

For peer review only

Author Contributions:

MJS: Conceived of the project, wrote the first draft, and contributed to the data analysis.

KB: Led the data analysis, contributed to manuscript production.

JOB: Helped conceive of the project and contributed to manuscript production.

DP: Contributed to data collection, project conception, and contributed to manuscript production.

OK: Contributed to data collection, project conception, and contributed to manuscript production.

EW: Contributed to data collection and project conception.

PM: Contributed to data collection, project conception, and contributed to manuscript production.

FT: Contributed to data collection and project conception.

KH: Contributed to data collection and project conception.

TB: Contributed to data collection, project conception, and contributed to manuscript production.

MB: Contributed to project conception, data analysis, and contributed to manuscript production.

All authors reviewed the manuscript for editorial contributions and approved of the final version.

Data Sharing:

Data for this analysis is available by emailing Mark Siedner (msiedner@mgh.harvard.edu) and Kobus Herbst (kherbst@ahri.org)

For peer review only

REFERENCES

1. Lozano R, Naghavi M, Foreman K, et al. Global and regional mortality from 235 causes of death for 20 age groups in 1990 and 2010: a systematic analysis for the Global Burden of Disease Study 2010. *Lancet* 2012;380(9859):2095-128. doi: 10.1016/S0140-6736(12)61728-0
2. Alwan A, Maclean DR, Riley LM, et al. Monitoring and surveillance of chronic non-communicable diseases: progress and capacity in high-burden countries. *Lancet* 2010;376(9755):1861-8. doi: 10.1016/S0140-6736(10)61853-3
3. Non-communicable Diseases Progress Monitor. World Health Organization. 2017. Avaialble at: https://ncdalliance.org/sites/default/files/resource_files/WHOProgressMonitor2017.pdf. Accessed 21st November 2017.
4. Global Action Plan for the Prevention and Control of Non-Communicable Diseases 2013-2020. World Health Organization. Available at: http://apps.who.int/iris/bitstream/10665/94384/1/9789241506236_eng.pdf. Accessed 21st November, 2017.
5. Yusuf S, Hawken S, Ounpuu S, et al. Effect of potentially modifiable risk factors associated with myocardial infarction in 52 countries (the INTERHEART study): case-control study. *Lancet* 2004;364(9438):937-52. doi: 10.1016/S0140-6736(04)17018-9 [published Online First: 2004/09/15]
6. O'Donnell MJ, Chin SL, Rangarajan S, et al. Global and regional effects of potentially modifiable risk factors associated with acute stroke in 32 countries (INTERSTROKE): a case-control study. *Lancet* 2016;388(10046):761-75. doi: 10.1016/S0140-6736(16)30506-2 [published Online First: 2016/07/20]
7. Health Systems Trust. South African Health Review, 2016.
8. Strategic Plan for the Prevention and Control of Non-Communicable Diseases 2013-17. South African Ministry of Health. Available at: http://www.hsrc.ac.za/uploads/pageContent/3893/NCDs STRAT PLAN_CONTENT 8 april proof.pdf Accessed 21st November 2017.
9. Chamie G, Kwarisiima D, Clark TD, et al. Leveraging rapid community-based HIV testing campaigns for non-communicable diseases in rural Uganda. *PLoS One* 2012;7(8):e43400. doi: 10.1371/journal.pone.0043400 [published Online First: 2012/08/24]
10. Gaziano TA, Abrahams-Gessel S, Denman CA, et al. An assessment of community health workers' ability to screen for cardiovascular disease risk with a simple, non-invasive risk assessment instrument in Bangladesh, Guatemala, Mexico, and South Africa: an observational study. *The Lancet Global health* 2015;3(9):e556-63. doi: 10.1016/S2214-109X(15)00143-6 [published Online First: 2015/07/19]
11. Tanser F, Hosegood V, Barnighausen T, et al. Cohort Profile: Africa Centre Demographic Information System (ACDIS) and population-based HIV survey. *International journal of epidemiology* 2008;37(5):956-62. doi: 10.1093/ije/dym211 [published Online First: 2007/11/14]
12. World Health Organization. Noncommunicable diseases and mental health cluster. WHO STEPS Surveillance Manual: The WHO STEPwise Approach to Chronic Disease Risk Factor Suveillance. Geneva. 2005
13. Malaza A, Mossong J, Barnighausen T, et al. Hypertension and obesity in adults living in a high HIV prevalence rural area in South Africa. *PLoS One* 2012;7(10):e47761. doi: 10.1371/journal.pone.0047761
14. Glanz K, Rimer BK, Viswanath K. Health behavior and health education: Theory, Research and Practice. 4th ed. San Francisco, CA: Jossey-Bass 2008.
15. Royston P, Ambler G, Sauerbrei W. The use of fractional polynomials to model continuous risk variables in epidemiology. *International journal of epidemiology* 1999;28(5):964-74. [published Online First: 1999/12/22]
16. van Rooyen H, Barnabas RV, Baeten JM, et al. High HIV testing uptake and linkage to care in a novel program of home-based HIV counseling and testing with facilitated referral in KwaZulu-Natal, South Africa. *J Acquir Immune Defic Syndr* 2013;64(1):e1-8. doi: 10.1097/QAI.0b013e31829b567d [published Online First: 2013/05/30]

For peer review only

17. Iwuji CC, Orne-Gliemann J, Larmarange J, et al. Universal test and treat and the HIV epidemic in rural South Africa: a phase 4, open-label, community cluster randomised trial. *Lancet HIV* 2017 doi: 10.1016/S2352-3018(17)30205-9 [published Online First: 2017/12/05]
18. Pastakia SD, Manyara SM, Vedanthan R, et al. Impact of Bridging Income Generation with Group Integrated Care (BIGPIC) on Hypertension and Diabetes in Rural Western Kenya. *J Gen Intern Med* 2017;32(5):540-48. doi: 10.1007/s11606-016-3918-5 [published Online First: 2016/12/07]
19. Musicha C, Crampin AC, Kayuni N, et al. Accessing clinical services and retention in care following screening for hypertension and diabetes among Malawian adults: an urban/rural comparison. *Journal of hypertension* 2016;34(11):2172-9. doi: 10.1097/HJH.0000000000001070 [published Online First: 2016/08/24]
20. Kotwani P, Balzer L, Kwarisiima D, et al. Evaluating linkage to care for hypertension after community-based screening in rural Uganda. *Trop Med Int Health* 2014;19(4):459-68. doi: 10.1111/tmi.12273 [published Online First: 2014/02/06]
21. Zack RM, Irema K, Kazonda P, et al. Determinants of high blood pressure and barriers to diagnosis and treatment in Dar es Salaam, Tanzania. *Journal of hypertension* 2016;34(12):2353-64. doi: 10.1097/HJH.0000000000001117 [published Online First: 2016/11/03]
22. Lankowski AJ, Siedner MJ, Bangsberg DR, et al. Impact of geographic and transportation-related barriers on HIV outcomes in sub-Saharan Africa: a systematic review. *AIDS Behav* 2014;18(7):1199-223. doi: 10.1007/s10461-014-0729-8
23. Siedner MJ, Lankowski A, Tsai AC, et al. GPS-measured distance to clinic, but not self-reported transportation factors, are associated with missed HIV clinic visits in rural Uganda. *AIDS* 2013;27(9):1503-8. doi: 10.1097/QAD.0b013e32835fd873
24. Tanser F, Gijsbertsen B, Herbst K. Modelling and understanding primary health care accessibility and utilization in rural South Africa: an exploration using a geographical information system. *Social science & medicine* 2006;63(3):691-705. doi: 10.1016/j.socscimed.2006.01.015 [published Online First: 2006/04/01]
25. Statistics South Africa. Gender statistics in South Africa, 2011. http://www.statssa.gov.za/publications/Report-03-10-05/Report-03-10-052011.pdf (accessed 27 February 2017). .
26. Sharma M, Ying R, Tarr G, et al. Systematic review and meta-analysis of community and facility-based HIV testing to address linkage to care gaps in sub-Saharan Africa. *Nature* 2015;528(7580):S77-85. doi: 10.1038/nature16044 [published Online First: 2015/12/04]
27. Krieger J, Collier C, Song L, et al. Linking community-based blood pressure measurement to clinical care: a randomized controlled trial of outreach and tracking by community health workers. *Am J Public Health* 1999;89(6):856-61. [published Online First: 1999/06/08]
28. Baig AA, Mangione CM, Sorrell-Thompson AL, et al. A randomized community-based intervention trial comparing faith community nurse referrals to telephone-assisted physician appointments for health fair participants with elevated blood pressure. *J Gen Intern Med* 2010;25(7):701-9. doi: 10.1007/s11606-010-1326-9 [published Online First: 2010/03/30]

For peer review only - http://bmjopen.bmj.com/site/about/guidelines.xhtml

Table 1. Participants with blood pressure measured in in 2010 survey, those with hypertension[1] and stratified by whether or not previously diagnosed or on treatment

	All participants	N with hypertension[1]	Previously diagnosed[2]	Not previously diagnosed[3]
	N=11,694	N=3074 (26.2%)	N=1368 (44.5%)	N=1706 (55.5%)
**Median (IQR) age (years)**	34 (21–53)	55 (43–68)	60 (51–71)	49 (35–63)
**Age group**				P<0.001[4]
<30	5107 (43.7%)	354 (11.5%)	21 (1.5 %)	333 (19.5%)
35-44	2191 (18.7%)	484 (15.7%)	118 (8.6 %)	366 (21.5%)
45-59	2265 (19.4%)	992 (32.3%)	507 (37.1%)	485 (28.4%)
60+	2131 (18.2%)	1244 (40.5%)	722 (52.8%)	522 (30.6%)
**Sex**				P<0.001
Male	3453 (29.5%)	720 (23.4%)	178 (13.0%)	542 (31.8%)
Female	8241 (70.5%)	2354 (76.6%)	1190 (87.0%)	1164 (68.2%)
**Education**				P<0.001
None	2389 (20.5%)	1032 (33.6%)	550 (40.2%)	482 (28.3%)
Less than complete secondary	6244 (53.5%)	1463 (47.6%)	662 (48.4%)	801 (47.0%)
Complete secondary/above	3040 (26.0%)	576 (18.8%)	155 (11.3%)	421 (24.7%)
Missing	*21*	*3*	*1*	*2*
**Marital status**				P<0.001
Single (never married)	3462 (29.8%)	518 (16.9%)	174 (12.7%)	344 (20.2%)
Married/informal union	6556 (56.3%)	1696 (55.2%)	688 (50.3%)	1008 (59.2%)
Widowed/separated/divorced	1618 (13.9%)	857 (27.9%)	506 (37.0%)	351 (20.6%)
Missing	*58*	*3*	*0*	*3*
**Employed**				P<0.001
Yes	1779 (15.3%)	437 (14.2%)	140 (10.2%)	297 (17.4%)
No	9828 (84.7%)	2634 (85.8%)	1228 (89.8%)	1406 (82.6%)
Missing	*87*	*3*	*0*	*3*
**Residence**				P<0.001
Urban	617 (5.3 %)	119 (3.9 %)	55 (4.0 %)	64 (3.8 %)
Peri-urban	3604 (30.8%)	904 (29.4%)	347 (25.4%)	557 (32.7%)
Rural	7464 (63.9%)	2050 (66.7%)	966 (70.6%)	1084 (63.6%)
Missing	*9*	*1*	*0*	*1*
**SES tertile**				P=0.67
Low	4193 (36.4%)	1173 (38.6%)	525 (38.7%)	648 (38.5%)
Middle	3818 (33.1%)	947 (31.2%)	412 (30.4%)	535 (31.8%)
High	3522 (30.5%)	918 (30.2%)	418 (30.8%)	500 (29.7%)
Missing	*161*	*36*	*13*	*23*
**Self-report of diabetes**[5]				P<0.001
No	11300 (96.6%)	2867 (93.3%)	1176 (86.0%)	1691 (99.1%)
Yes	394 (3.4 %)	207 (6.7 %)	192 (14.0%)	15 (0.9 %)
**Nearest clinic (km)** [6]				P=0.84
0- <1.5	2642 (22.6%)	676 (22.0%)	292 (21.3%)	384 (22.5%)
1.5-2.5	2879 (24.6%)	710 (23.1%)	314 (23.0%)	396 (23.2%)
>2.5-3.9	2975 (25.5%)	809 (26.3%)	368 (26.9%)	441 (25.9%)
>3.9	3189 (27.3%)	878 (28.6%)	394 (28.8%)	484 (28.4%)
Missing	*9*	*1*	*0*	*1*

[1]Hypertension defined as systolic BP ≥ 140mmHg or diastolic BP ≥90 mmHg, in an average of 2 readings. [2]Report having been previously diagnosed or on treatment for hypertension in 2010 survey. [3]Report no previous diagnosis or treatment for hypertension in 2010 survey. [4]P-value from Chi-squared test comparing characteristics of those previously diagnosed/treatment and those with no previous diagnosis/treatment. [5]Report having been diagnosed with or on treatment for diabetes in 2010 survey. [6]Quartiles based on distribution in all individuals who were eligible for 2010 survey.

For peer review only

Table 2. Factors[1] associated with linkage to hypertension care within 2 years after home-based diagnosis of hypertension[2] in 2010, among individuals who were previously undiagnosed and participated in 2011 or 2012 (N=1199) (unweighted analysis)

	Linked to care/N (%)	Crude OR (95% CI)	Age- & sex-adjusted OR (95% CI)	Adjusted OR (95% CI)[3]
**Sociodemographic factors**				
**Age group**		P<0.001	P<0.001	P<0.001
<30	10 / 193 (5.2 %)	1	1	1
35-44	40 / 242 (16.5%)	3.62 (1.76 -7.45)	3.08 (1.49 -6.36)	3.32 (1.60 -6.89)
45-59	126 / 360 (35.0%)	9.85 (5.03 -19.30)	8.39 (4.26 -16.51)	9.01 (4.57 -17.79)
60+	167 / 404 (41.3%)	12.89 (6.62 -25.11)	11.61 (5.94 -22.69)	11.49 (5.87 -22.46)
**Sex**		P<0.001	P<0.001	P<0.001
Male	47 / 308 (15.3%)	1	1	1
Female	296 / 891 (33.2%)	2.76 (1.97 -3.88)	2.50 (1.75 -3.57)	2.41 (1.68 -3.45)
**Marital status**		P<0.001	P=0.15	P=0.14
Single (never married)	55 / 250 (22.0%)	1	1	1
Married/informal union	178 / 666 (26.7%)	1.29 (0.92 -1.83)	1.33 (0.91 -1.95)	1.35 (0.92 -1.98)
Widow/sep/divorced	110 / 283 (38.9%)	2.25 (1.54 -3.31)	0.98 (0.64 -1.50)	0.99 (0.65 -1.51)
**Education**		P<0.001	P=0.83	P=0.77
None	132 / 363 (36.4%)	1	1	1
Less than complete secondary	160 / 581 (27.5%)	0.67 (0.50 -0.88)	1.10 (0.81 -1.49)	1.09 (0.80 -1.49)
Complete secondary/above	51 / 255 (20.0%)	0.44 (0.30 -0.64)	1.09 (0.71 -1.67)	1.15 (0.75 -1.78)
**Employed**		P<0.001	P=0.02	P=0.02
Yes	31 / 178 (17.4%)	1	1	1
No	312 / 1021 (30.6%)	2.09 (1.39 -3.14)	1.71 (1.10 -2.65)	1.71 (1.10 -2.65)
**SES tertile**		P=0.307	P=0.31	P=0.21
Low	125 / 459 (27.2%)	1	1	1
Middle	99 / 364 (27.2%)	1.00 (0.73 -1.36)	1.09 (0.79 -1.52)	1.12 (0.81 -1.56)
High	115 / 364 (31.6%)	1.23 (0.91 -1.67)	1.28 (0.93 -1.77)	1.34 (0.97 -1.85)
**Location factors**				
**Residence**		P=0.04	P=0.35	P=0.55
Urban	10 / 36 (27.8%)	1	1	1
Peri-urban	95 / 398 (23.9%)	0.82 (0.38 -1.75)	0.75 (0.33 -1.69)	0.67 (0.29 -1.53)
Rural	238 / 765 (31.1%)	1.17 (0.56 -2.47)	0.92 (0.41 -2.05)	0.63 (0.27 -1.45)
**Nearest clinic (km)** [4]				
0- <1.5	56 / 263 (21.3%)			
1.5-2.5	71 / 269 (26.4%)	P<0.001	P=0.001	P=0.001
>2.5-3.9	93 / 310 (30.0%)	1.15 (1.08 -1.23)[5]	1.13 (1.05 -1.21)[5]	1.12 (1.05 -1.20)[5]
>3.9	123 / 357 (34.5%)			
**Clinical factors**				
**BMI category**		P<0.001	P=0.07	P=0.13
<25 kg/m^2	70 / 344 (20.3%)	1	1	1
25 – <30 kg/m^2	62 / 229 (27.1%)	1.45 (0.98 -2.15)	1.08 (0.71 -1.64)	1.17 (0.76 -1.81)
≥30 kg/m^2	110 / 301 (36.5%)	2.25 (1.59 -3.21)	1.52 (1.03 -2.24)	1.51 (1.00 -2.26)
**Hypertension stage**[6]		P<0.001	P<0.001	P<0.001
Stage I	142 / 730 (19.5%)	1	1	1
Stage II	134 / 342 (39.2%)	2.67 (2.01 -3.54)	2.22 (1.65 -2.99)	2.20 (1.63 -2.97)
Hypertension urgency	67 / 127 (52.8%)	4.62 (3.12 -6.85)	3.12 (2.06 -4.74)	3.07 (2.01 -4.67)
**Self-report of diabetes**[7]		P=0.19	P=0.44	P=0.47
No	339 / 1191 (28.5%)	1	1	1
Yes	4 / 8 (50.0%)	2.51 (0.62 -10.11)	1.78 (0.41 -7.70)	1.75 (0.38 -8.15)

For peer review only

For peer review only - http://bmjopen.bmj.com/site/about/guidelines.xhtml

**Self-report of TB[7]**		P=0.66	P=0.96	P=0.72
No	332 / 1156 (28.7%)	1	1	1
Yes	11 / 43 (25.6%)	0.85 (0.43 -1.71)	1.02 (0.49 -2.13)	1.15 (0.54 -2.46)

[1]All characteristics are based on 2010 survey. [2]Hypertension defined as systolic BP ≥ 140mmHg or diastolic BP ≥90 mmHg, in an average of 2 readings. [3]Sociodemographic factors adjusted for age group, sex, and employment. Location factors adjusted for age group, sex, employment, and distance from nearest clinic as continuous covariate. Clinical factors adjusted for age group, sex, employment, distance from nearest clinic and hypertension stage. [4]Quartiles based on distribution in all individuals who were eligible for 2010 survey. Fit as continuous covariate; n (%) linked in each distance quartile shown for information only. [5]OR for linear trend in linkage with each 1 km increase in distance. [6]Stage I: Systolic BP 140–159 or diastolic BP 90–99; Stage II: Systolic BP 160–179 or diastolic BP 100–119; Hypertension urgency: Systolic BP ≥180 or diastolic BP ≥120. [7]Reports being diagnosed in the past 12m or currently on treatment

For peer review only

Figure 1. Flow diagram of eligible and included participants in a baseline community-based hypertension screen in 2010 and follow-up observation during 2011/2012

For peer review only

Figure 2. Proportion of individuals linked to hypertensive care two years after a new notification of elevated blood pressure (weighted estimates)

For peer review only

For peer review only

For peer review only - http://bmjopen.bmj.com/site/about/guidelines.xhtml

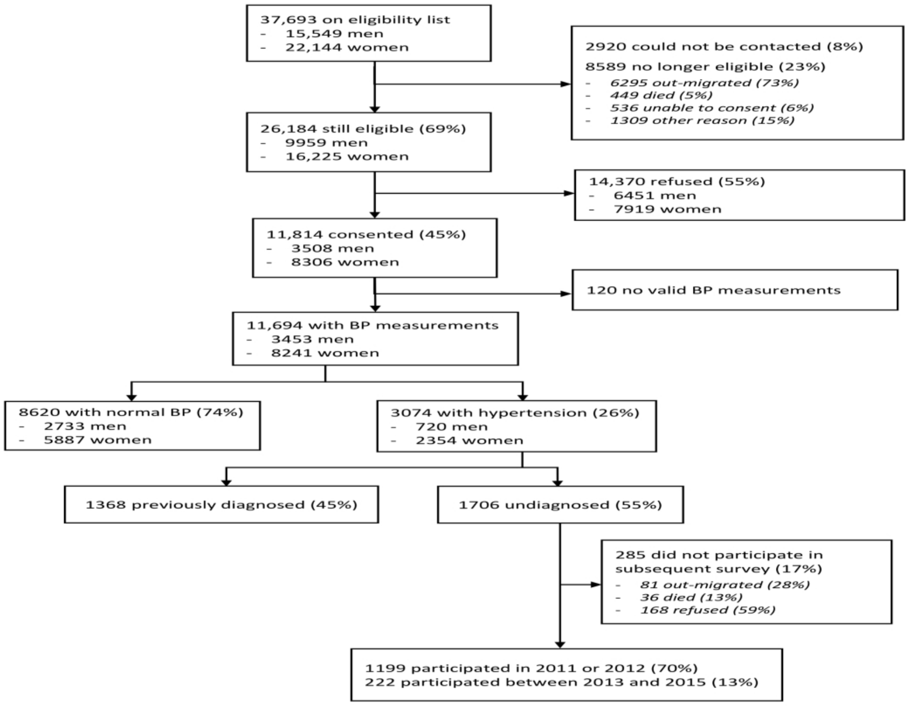

Figure 1. Flow diagram of eligible and included participants in a baseline community-based hypertension screen in 2010 and follow-up observation during 2011/2012

1057x793mm (72 x 72 DPI)

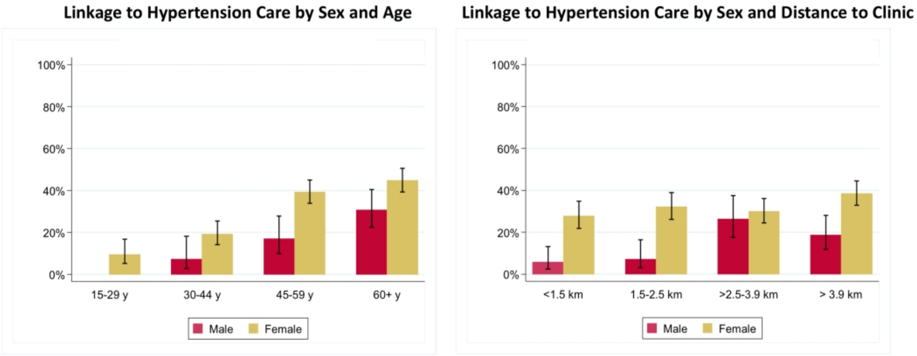

Figure 2. Proportion of individuals linked to hypertensive care two years after a new notification of elevated blood pressure (weighted estimates)

1057x595mm (72 x 72 DPI)

Supplementary Table 1. Comparison of characteristics between those for whom a blood pressure measurement was taken in the 2010 survey, and those with no blood pressure measurements

	**Eligible for survey[1]**	**BP measurements**	**No BP measurement[2]**
	**N=26,184**	**N=11,694**	**N=14,490**
**Median (IQR) age (years)**	32 (20–50)	34 (21–53)	30 (20–47)
**Age group**			**P<0.001[3]**
<30	12,164 (46.5%)	5106 (43.7%)	7058 (48.7%)
35-44	5523 (21.1%)	2194 (18.8%)	3329 (23.0%)
45-59	4584 (17.5%)	2251 (19.2%)	2333 (16.1%)
60+	3913 (14.9%)	2143 (18.3%)	1770 (12.2%)
**Sex**			**P<0.001**
Male	9959 (38.0%)	3453 (29.5%)	6506 (44.9%)
Female	16,225 (62.0%)	8241 (70.5%)	7984 (55.1%)
**Marital status**			**P<0.001**
Single (never married)	8113 (31.2%)	3462 (29.8%)	4651 (32.4%)
Married/informal union	14,959 (57.6%)	6556 (56.3%)	8403 (58.6%)
Widow/sep/divorced	2910 (11.2%)	1618 (13.9%)	1292 (9.0 %)
Missing	*202*	*58*	*144*
**Education**			**P<0.001**
None	4558 (17.5%)	2389 (20.5%)	2169 (15.1%)
Less than complete secondary	13,466 (51.6%)	6244 (53.5%)	7222 (50.1%)
Complete secondary/above	8051 (30.9%)	3040 (26.0%)	5011 (34.8%)
Missing	*109*	*21*	*88*
**Employed**			**P<0.001**
Yes	5860 (22.7%)	1779 (15.3%)	4081 (28.6%)
No	19,997 (77.3%)	9828 (84.7%)	10,169 (71.4%)
Missing	*327*	*87*	*240*
**Residence**			**P<0.001**
Urban	1953 (7.5 %)	617 (5.3 %)	1336 (9.2 %)
Peri-urban	8084 (30.9%)	3604 (30.8%)	4480 (31.0%)
Rural	16,092 (61.6%)	7464 (63.9%)	8628 (59.7%)
Missing	*55*	*9*	*46*
**SES tertile**			**P<0.001**
Low	8566 (33.6%)	4193 (36.4%)	4373 (31.4%)
Middle	8330 (32.7%)	3818 (33.1%)	4512 (32.4%)
High	8569 (33.7%)	3522 (30.5%)	5047 (36.2%)
Missing	*719*	*161*	*558*

[1]Individuals who were on the eligibility list for the 2010 survey (aged ≥15 years as of Dec 2009 and resident in the DSS), were successfully contacted (92% of all on the list) and still eligible at the time of contact (75% of those contacted). [2]Includes 14,370 individuals who refused consent, and 120 individuals who consented but for whom blood pressure measurements were not available. [3]P-value from Chi-squared test comparing those with blood pressure measurements and those without.

For peer review only

Supplementary Table 2. Comparison of characteristics between those who participated in the general health survey in 2011 or 2012 and those who did not, among 1706 individuals with undiagnosed hypertension in 2010

	Participated in 2011/2012	Did not participate in 2011/2012	Participated in 2011–2016	No participation in later survey
	**N=1199 (70.3%)**	**N=507 (29.7%)**	**N=1421 (83.3%)**	**N=285 (16.7%)**
**Median (IQR) age (years)**	50 (38–66)	43 (29–58)	50 (37–65)	41 (28–57)
**Age group**		P<0.001[1]		P<0.001[2]
<30	193 (16.1%)	140 (27.6%)	248 (17.5%)	85 (29.8%)
35-44	242 (20.2%)	124 (24.5%)	284 (20.0%)	82 (28.8%)
45-59	360 (30.0%)	125 (24.7%)	430 (30.3%)	55 (19.3%)
60+	404 (33.7%)	118 (23.3%)	459 (32.3%)	63 (22.1%)
**Sex**		P<0.001		P<0.001
Male	308 (25.7%)	234 (46.2%)	394 (27.7%)	148 (51.9%)
Female	891 (74.3%)	273 (53.8%)	1027 (72.3%)	137 (48.1%)
**Marital status**		P<0.001		P<0.001
Single (never married)	250 (20.9%)	94 (18.7%)	287 (20.2%)	57 (20.2%)
Married/informal union	666 (55.5%)	342 (67.9%)	817 (57.5%)	191 (67.7%)
Widow/sep/divorced	283 (23.6%)	68 (13.5%)	317 (22.3%)	34 (12.1%)
**Education**		P<0.001		P<0.001
None	363 (30.3%)	119 (23.6%)	424 (29.8%)	58 (20.5%)
Less than complete secondary	581 (48.5%)	220 (43.6%)	678 (47.7%)	123 (43.5%)
Complete secondary/above	255 (21.3%)	166 (32.9%)	319 (22.4%)	102 (36.0%)
**Employed**		P<0.001		P<0.001
Yes	178 (14.8%)	119 (23.6%)	220 (15.5%)	77 (27.3%)
No	1021 (85.2%)	385 (76.4%)	1201 (84.5%)	205 (72.7%)
**Residence**		P=0.04		P=0.16
Urban	36 (3.0 %)	28 (5.5 %)	48 (3.4 %)	16 (5.6 %)
Peri-urban	398 (33.2%)	159 (31.4%)	470 (33.1%)	87 (30.6%)
Rural	765 (63.8%)	319 (63.0%)	903 (63.5%)	181 (63.7%)
**SES tertile**		P=0.24		P=0.05
Low	459 (38.7%)	189 (38.1%)	544 (38.7%)	104 (37.5%)
Middle	364 (30.7%)	171 (34.5%)	431 (30.7%)	104 (37.5%)
High	364 (30.7%)	136 (27.4%)	431 (30.7%)	69 (24.9%)
**BMI category**		P=0.008		P<0.001
Underweight	50 (5.7 %)	25 (7.2 %)	58 (5.6 %)	17 (9.0 %)
Normal weight	294 (33.6%)	146 (42.3%)	351 (34.0%)	89 (47.3%)
Overweight	229 (26.2%)	84 (24.3%)	269 (26.1%)	44 (23.4%)
Obese	301 (34.4%)	90 (26.1%)	353 (34.2%)	38 (20.2%)
**Distance to nearest clinic (km)**		P=0.22		P=0.50
0- <1.5	263 (21.9%)	121 (23.9%)	320 (22.5%)	64 (22.5%)
1.5-2.5	269 (22.4%)	127 (25.1%)	323 (22.7%)	73 (25.7%)
>2.5-3.9	310 (25.9%)	131 (25.9%)	365 (25.7%)	76 (26.8%)
>3.9	357 (29.8%)	127 (25.1%)	413 (29.1%)	71 (25.0%)

[1]P-value from Chi-squared test comparing individuals who participated in 2011-2012 (N=1199) with those who did not (N=507). [2]P-value from Chi-squared test comparing individuals who participated in 2011-2015 (N=1421) with those who did not (N=285).

For peer review only

For peer review only - http://bmjopen.bmj.com/site/about/guidelines.xhtml

Supplementary Table 3. Factors[1] associated with linkage to hypertension care within 2 years after home-based diagnosis of hypertension[2] in 2010, among individuals who were previously undiagnosed and participated in 2011 or 2012 (N=1199), using inverse probability weighting to account for non-participation in the blood pressure screen

	Linked to care/N (%)	Crude OR (95% CI)[3]	Age- & sex-adjusted OR (95% CI)[3]	Adjusted OR (95% CI)[3,4]
**Sociodemographic factors**				
**Age group**		P<0.001	P<0.001	P<0.001
<30	10 / 193 (5.2 %)	1	1	1
35-44	40 / 242 (16.5%)	3.82 (1.85 -7.87)	3.20 (1.55 -6.62)	3.26 (1.54 -6.90)
45-59	126 / 360 (35.0%)	10.35 (5.28 -20.32)	8.64 (4.39 -16.99)	9.35 (4.73 -18.46)
60+	167 / 404 (41.3%)	13.75 (7.05 -26.81)	12.27 (6.29 -23.91)	13.48 (6.82 -26.61)
**Sex**		P<0.001	P<0.001	P<0.001
Male	47 / 308 (15.3%)	1	1	1
Female	296 / 891 (33.2%)	2.86 (2.03 -4.03)	2.57 (1.81 -3.66)	2.77 (1.91 -4.00)
**Marital status**		P<0.001	P=0.09	P=0.09
Single (never married)	55 / 250 (22.0%)	1	1	1
Married/informal union	178 / 666 (26.7%)	1.35 (0.95 -1.91)	1.35 (0.91 -1.99)	1.36 (0.92 -2.01)
Widow/sep/divorced	110 / 283 (38.9%)	2.40 (1.63 -3.54)	0.94 (0.61 -1.44)	0.95 (0.61 -1.45)
**Education**		P<0.001	P=0.86	P=0.89
None	132 / 363 (36.4%)	1	1	1
Less than complete secondary	160 / 581 (27.5%)	0.64 (0.48 -0.84)	1.09 (0.80 -1.49)	1.07 (0.78 -1.46)
Complete secondary/above	51 / 255 (20.0%)	0.42 (0.29 -0.61)	1.06 (0.69 -1.63)	1.10 (0.71 -1.70)
**Employed**		P<0.001	P=0.01	P=0.01
Yes	31 / 178 (17.4%)	1	1	1
No	312 / 1021 (30.6%)	2.21 (1.46 -3.34)	1.74 (1.13 -2.69)	1.76 (1.14 -2.72)
**SES tertile**		P=0.36	P=0.32	P=0.26
Low	125 / 459 (27.2%)	1	1	1
Middle	99 / 364 (27.2%)	1.01 (0.74 -1.38)	1.11 (0.79 -1.55)	1.12 (0.81 -1.57)
High	115 / 364 (31.6%)	1.23 (0.90 -1.66)	1.28 (0.93 -1.77)	1.31 (0.95 -1.82)
**Location factors**				
**Residence**		P=0.03	P=0.40	P=0.68
Urban	10 / 36 (27.8%)	1	1	1
Peri-urban	95 / 398 (23.9%)	0.91 (0.42 -1.96)	0.84 (0.37 -1.89)	0.73 (0.32 -1.67)
Rural	238 / 765 (31.1%)	1.32 (0.62 -2.79)	1.03 (0.47 -2.27)	0.69 (0.30 -1.59)
**Nearest clinic (km)** [5]				
0- <1.5	56 / 263 (21.3%)			
1.5-2.5	71 / 269 (26.4%)	P<0.001	P=0.001	P=0.002
>2.5-3.9	93 / 310 (30.0%)	1.15 (1.08 -1.23) [6]	1.13 (1.05 -1.21) [6]	1.12 (1.04 -1.20) [6]
>3.9	123 / 357 (34.5%)			
**Clinical factors**				
**BMI category**		P<0.001	P=0.06	P=0.14
<25 kg/m^2	70 / 344 (20.3%)	1	1	1
25 – <30 kg/m^2	62 / 229 (27.1%)	1.56 (1.05 -2.33)	1.13 (0.73 -1.74)	1.19 (0.77 -1.84)
≥30 kg/m^2	110 / 301 (36.5%)	2.42 (1.69 -3.45)	1.57 (1.05 -2.34)	1.51 (1.00 -2.28)
**Hypertension stage**[7]		P<0.001	P<0.001	P<0.001
Stage I	142 / 730 (19.5%)	1	1	1
Stage II	134 / 342 (39.2%)	2.72 (2.04 -3.63)	2.20 (1.62 -2.99)	2.15 (1.58 -2.93)
Hypertension urgency	67 / 127 (52.8%)	4.79 (3.22 -7.13)	3.14 (2.06 -4.78)	3.10 (2.04 -4.71)
**Self-report of diabetes**[8]		P=0.28	P=0.50	P=0.56
No	339 / 1191 (28.5%)	1	1	1
Yes	4 / 8 (50.0%)	2.16 (0.53 -8.83)	1.56 (0.43 -5.65)	1.48 (0.40 -5.52)

For peer review only

**Self-report of TB[8]**		P=0.81	P=0.77	P=0.48
No	332 / 1156 (28.7%)	1	1	1
Yes	11 / 43 (25.6%)	0.92 (0.45 -1.86)	1.12 (0.51 -2.49)	1.34 (0.59 -3.04)

[1]All characteristics are based on 2010 survey. [2]Hypertension defined as systolic BP ≥ 140mmHg or diastolic BP ≥90 mmHg, in an average of 2 readings. [3]Weighted for non-response; weights calculated as the inverse probability of survey participation, in strata defined by age group, sex, education level and place of residence. [4]Sociodemographic factors adjusted for age group, sex, marital status and employment. Location factors adjusted for age group, sex, marital status, employment, and distance from nearest clinic as continuous covariate. Clinical factors adjusted for age group, sex, marital status, employment, distance from nearest clinic, and hypertension stage. [5]Quartiles based on distribution in all individuals who were eligible for 2010 survey. Fit as continuous covariate; n (%) linked in each distance quartile shown for information only. [6]OR for linear trend in linkage with each 1 km increase in distance. [7]Stage I: Systolic BP 140–159 or diastolic BP 90–99; Stage II: Systolic BP 160–179 or diastolic BP 100–119; Hypertension urgency: Systolic BP ≥180 or diastolic BP ≥120. [8]Reports being diagnosed in the past 12m or currently on treatment.

For peer review only

Supplementary Table 4. Factors[1] associated with linkage to hypertension care within 5 years (2011 to 2015) after home-based diagnosis of hypertension[2] in 2010, among individuals who were previously undiagnosed and participated in a subsequent survey (N=1421) using inverse probability weighting to account for non-participation in the blood pressure screen

	Linked to care/N (%)	Crude OR (95% CI)[3]	Age- & sex-adjusted OR (95% CI)[3]	Adjusted OR (95% CI)[3,4]
**Sociodemographic factors**				
**Age group**		P<0.001	P<0.001	P<0.001
<30	25 / 248 (10.1%)	1	1	1
35-44	75 / 284 (26.4%)	3.22 (1.97 -5.29)	2.69 (1.64 -4.43)	2.93 (1.77 -4.87)
45-59	211 / 430 (49.1%)	8.67 (5.48 -13.70)	7.50 (4.73 -11.89)	8.05 (5.05 -12.84)
60+	265 / 459 (57.7%)	12.37 (7.84 -19.53)	11.65 (7.35 -18.47)	11.49 (7.25 -18.22)
**Sex**		P<0.001	P<0.001	P<0.001
Male	92 / 394 (23.4%)	1	1	1
Female	484 / 1027 (47.1%)	3.06 (2.35 -3.99)	2.98 (2.25 -3.94)	2.85 (2.15 -3.79)
**Marital status**		P<0.001	P=0.66	P=0.64
Single (never married)	98 / 287 (34.1%)	1	1	1
Married/informal union	298 / 817 (36.5%)	1.16 (0.88 -1.55)	1.04 (0.76 -1.44)	1.07 (0.77 -1.48)
Widow/sep/divorced	180 / 317 (56.8%)	2.78 (1.99 -3.87)	0.90 (0.61 -1.32)	0.92 (0.62 -1.35)
**Education**		P<0.001	P=0.55	P=0.66
None	217 / 424 (51.2%)	1	1	1
Less than complete secondary	269 / 678 (39.7%)	0.60 (0.47 -0.76)	1.13 (0.85 -1.50)	1.13 (0.86 -1.50)
Complete secondary/above	90 / 319 (28.2%)	0.36 (0.26 -0.49)	0.98 (0.68 -1.41)	1.05 (0.73 -1.53)
**Employed**		P<0.001	P=0.007	P=0.007
Yes	61 / 220 (27.7%)	1	1	1
No	515 / 1201 (42.9%)	2.04 (1.48 -2.81)	1.62 (1.14 -2.29)	1.62 (1.14 -2.29)
**SES tertile**		P=0.49	P=0.71	P=0.58
Low	224 / 544 (41.2%)	1	1	1
Middle	163 / 431 (37.8%)	0.89 (0.68 -1.15)	0.95 (0.71 -1.27)	0.97 (0.73 -1.30)
High	183 / 431 (42.5%)	1.04 (0.80 -1.35)	1.08 (0.81 -1.43)	1.13 (0.85 -1.50)
**Location factors**				
**Residence**		P=0.07	P=0.89	P=0.61
Urban	18 / 48 (37.5%)	1	1	1
Peri-urban	173 / 470 (36.8%)	1.03 (0.56 -1.92)	0.95 (0.50 -1.79)	0.84 (0.44 -1.62)
Rural	385 / 903 (42.6%)	1.34 (0.73 -2.46)	1.01 (0.54 -1.88)	0.75 (0.39 -1.46)
**Nearest clinic (km)** [5]				
0- <1.5	111 / 320 (34.7%)			
1.5-2.5	128 / 323 (39.6%)	P=0.001	P=0.03	P=0.05
>2.5-3.9	147 / 365 (40.3%)	1.10 (1.04 -1.17)[6]	1.08 (1.01 -1.15)[6]	1.07 (1.00 -1.14)[6]
>3.9	190 / 413 (46.0%)			
**BMI category**		P<0.001	P=0.004	P=0.008
<25 kg/m^2	133 / 409 (32.5%)	1	1	1
25 – <30 kg/m^2	99 / 269 (36.8%)	1.28 (0.92 -1.77)	0.88 (0.61 -1.26)	0.90 (0.62 -1.31)
≥30 kg/m^2	186 / 353 (52.7%)	2.44 (1.81 -3.29)	1.55 (1.10 -2.18)	1.53 (1.08 -2.17)
**Hypertension stage**[7]		P<0.001	P<0.001	P<0.001
Stage I	264 / 863 (30.6%)	1	1	1
Stage II	215 / 415 (51.8%)	2.49 (1.95 -3.17)	2.01 (1.54 -2.62)	2.12 (1.55 -2.89)
Hypertension urgency	97 / 143 (67.8%)	5.02 (3.42 -7.37)	3.18 (2.10 -4.82)	3.29 (2.01 -5.39)
**Self-report of diabetes**[8]		P=0.05	P=0.08	P=0.32
No	568 / 1410 (40.3%)	1	1	1
Yes	8 / 11 (72.7%)	3.84 (0.98 -15.03)	3.09 (0.88 -10.81)	5.18 (0.21 -128.15)

For peer review only

For peer review only - http://bmjopen.bmj.com/site/about/guidelines.xhtml

**Self-report of TB[8]**		P=0.73	P=0.86	P=0.30
No	560 / 1376 (40.7%)	1	1	1
Yes	16 / 45 (35.6%)	0.89 (0.48 -1.68)	1.07 (0.49 -2.33)	1.57 (0.67 -3.71)

[1]All characteristics are based on 2010 survey. [2]Hypertension defined as systolic BP ≥ 140mmHg or diastolic BP ≥90 mmHg, in an average of 2 readings. [3]Weighted for non-response; weights calculated as the inverse probability of survey participation, in strata defined by age group, sex, education level and place of residence.
[4]Sociodemographic factors adjusted for age group, sex, and employment. Location factors adjusted for age group, sex, employment, and distance from nearest clinic as continuous covariate. Clinical factors adjusted for age group, sex, employment, distance from nearest clinic, hypertension stage, and BMI. [5]Quartiles based on distribution in all individuals who were eligible for 2010 survey. Fit as continuous covariate; n (%) linked in each distance quartile shown for information only. [6]OR for linear trend in linkage with each 1 km increase in distance. [7]Stage I: Systolic BP 140–159 or diastolic BP 90–99; Stage II: Systolic BP 160–179 or diastolic BP 100–119; Hypertension urgency: Systolic BP ≥180 or diastolic BP ≥120. [8]Reports being diagnosed in the past 12m or currently on treatment.

For peer review only

Supplementary Table 5. Factors[1] associated with linkage to hypertension care within 5 years (2011 to 2015) after home-based diagnosis of hypertension[2] in 2010, among individuals who were previously undiagnosed and participated in a subsequent survey (N=1421)

	Linked to care/N (%)	Crude OR (95% CI)[3]	Adjusted OR (95% CI)[3,4]	Adjusted OR (95% CI)[3,5]
**Sociodemographic factors**				
**Age group**		P<0.001	P<0.001	P<0.001
<30	25 / 248 (10.1%)	1	1	1
35-44	75 / 284 (26.4%)	3.22 (1.97 -5.29)	2.43 (1.47 -4.01)	2.61 (1.56 -4.34)
45-59	211 / 430 (49.1%)	8.67 (5.48 -13.70)	6.58 (4.13 -10.47)	6.96 (4.35 -11.14)
60+	265 / 459 (57.7%)	12.37 (7.84 -19.53)	10.23 (6.44 -16.25)	10.11 (6.37 -16.06)
**Sex**		P<0.001	P<0.001	P<0.001
Male	92 / 394 (23.4%)	1	1	1
Female	484 / 1027 (47.1%)	3.06 (2.35 -3.99)	2.54 (1.91 -3.39)	2.46 (1.84 -3.30)
**Times participated**				
Once	61 / 321 (19.0%)			
Twice	119 / 335 (35.5%)	P<0.001	P<0.001	P<0.001
3 times	160 / 327 (48.9%)	1.57 (1.44 -1.71)[6]	1.40 (1.27 -1.53)[6]	1.39 (1.26 -1.52)[6]
4 times	147 / 287 (51.2%)			
5 times	89 / 151 (58.9%)			
**Marital status**		P<0.001	P=0.60	P=0.55
Single (never married)	98 / 287 (34.1%)	1	1	1
Married/informal union	298 / 817 (36.5%)	1.16 (0.88 -1.55)	1.14 (0.82 -1.60)	1.16 (0.83 -1.63)
Widow/sep/divorced	180 / 317 (56.8%)	2.78 (1.99 -3.87)	1.00 (0.67 -1.48)	1.01 (0.68 -1.50)
**Education**		P<0.001	P=0.74	P=0.67
None	217 / 424 (51.2%)	1	1	1
Less than complete secondary	269 / 678 (39.7%)	0.60 (0.47 -0.76)	1.12 (0.84 -1.49)	1.12 (0.84 -1.50)
Complete secondary/above	90 / 319 (28.2%)	0.36 (0.26 -0.49)	1.09 (0.75 -1.59)	1.16 (0.79 -1.69)
**Employed**		P<0.001	P=0.03	P=0.03
Yes	61 / 220 (27.7%)	1	1	1
No	515 / 1201 (42.9%)	2.04 (1.48 -2.81)	1.48 (1.04 -2.10)	1.48 (1.04 -2.10)
**SES tertile**		P=0.49	P=0.68	P=0.56
Low	224 / 544 (41.2%)	1	1	1
Middle	163 / 431 (37.8%)	0.89 (0.68 -1.15)	0.98 (0.73 -1.32)	1.00 (0.74 -1.35)
High	183 / 431 (42.5%)	1.04 (0.80 -1.35)	1.11 (0.83 -1.48)	1.15 (0.86 -1.54)
**Location factors**				
**Residence**		P=0.07	P=0.63	P=0.61
Urban	18 / 48 (37.5%)	1	1	1
Peri-urban	173 / 470 (36.8%)	1.03 (0.56 -1.92)	0.82 (0.43 -1.57)	0.76 (0.40 -1.48)
Rural	385 / 903 (42.6%)	1.34 (0.73 -2.46)	0.93 (0.49 -1.74)	0.85 (0.45 -1.61)
**Nearest clinic (km)**[7]				
0- <1.5	111 / 320 (34.7%)			
1.5-2.5	128 / 323 (39.6%)	P=0.001	P=0.06	P=0.10
>2.5-3.9	147 / 365 (40.3%)	1.10 (1.04 -1.17)[8]	1.07 (1.00 -1.14)[8]	1.06 (0.99 -1.13)[8]
>3.9	190 / 413 (46.0%)			
**BMI category**		P<0.001	P=0.003	P=0.007
<25 kg/m^2	133 / 409 (32.5%)	1	1	1
25 – <30 kg/m^2	99 / 269 (36.8%)	1.28 (0.92 -1.77)	0.88 (0.61 -1.28)	0.89 (0.61 -1.30)
≥30 kg/m^2	186 / 353 (52.7%)	2.44 (1.81 -3.29)	1.58 (1.11 -2.25)	1.54 (1.07 -2.22)
**Hypertension stage**[9]		P<0.001	P<0.001	P<0.001
Stage I	264 / 863 (30.6%)	1	1	1
Stage II	215 / 415 (51.8%)	2.49 (1.95 -3.17)	2.19 (1.67 -2.88)	2.36 (1.71 -3.26)
Hypertension urgency	97 / 143 (67.8%)	5.02 (3.42 -7.37)	3.44 (2.25 -5.25)	3.74 (2.25 -6.20)

For peer review only

For peer review only - http://bmjopen.bmj.com/site/about/guidelines.xhtml

**Self-report of diabetes[10]**		P=0.05	P=0.03	P=0.28
No	568 / 1410 (40.3%)	1	1	1
Yes	8 / 11 (72.7%)	3.84 (0.98 -15.03)	3.67 (1.14 -11.80)	5.64 (0.25 -126.50)
**Self-report of TB[10]**		P=0.73	P=0.91	P=0.56
No	560 / 1376 (40.7%)	1	1	1
Yes	16 / 45 (35.6%)	0.89 (0.48 -1.68)	0.96 (0.43 -2.13)	1.31 (0.52 -3.27)

[1]All characteristics are based on 2010 survey. [2]Hypertension defined as systolic BP ≥ 140mmHg or diastolic BP ≥90 mmHg, in an average of 2 readings. [3]Weighted for non-response; weights calculated as the inverse probability of survey participation, in strata defined by age group, sex, education level and place of residence. [4]Adjusted for age, sex and number of times participated in subsequent surveys as a continuous covariate. [5]Sociodemographic factors adjusted for age group, sex, number of times in subsequent surveys, and employment. Location factors adjusted for age group, sex, number of times in subsequent surveys, and employment. Clinical factors adjusted for age group, sex, number of times in subsequent surveys, employment, hypertension stage, and BMI. [6]OR for linear trend in reported linkage with each unit increase in survey participation. [7]Quartiles based on distribution in all individuals who were eligible for 2010 survey. Fit as continuous covariate; n (%) linked in each distance quartile shown for information only. [8]OR for linear trend in linkage with each 1 km increase in distance. [9]Stage I: Systolic BP 140–159 or diastolic BP 90–99; Stage II: Systolic BP 160–179 or diastolic BP 100–119; Hypertension urgency: Systolic BP ≥180 or diastolic BP ≥120. [10]Reports being diagnosed in the past 12m or currently on treatment.

For peer review only

STROBE Statement—Checklist of items that should be included in reports of ***cohort studies***

	Item No	Recommendation	Page	Comments
**Title and abstract**	1	(*a*) Indicate the study's design with a commonly used term in the title or the abstract	1	
		(*b*) Provide in the abstract an informative and balanced summary of what was done and what was found	2	
**Introduction**				
Background/rationale	2	Explain the scientific background and rationale for the investigation being reported	4	Lines 2-23
Objectives	3	State specific objectives, including any prespecified hypotheses	4-5	Lines 25-32
**Methods**				
Study design	4	Present key elements of study design early in the paper	5	Lines 36-43
Setting	5	Describe the setting, locations, and relevant dates, including periods of recruitment, exposure, follow-up, and data collection	5	Lines 36-43
Participants	6	(*a*) Give the eligibility criteria, and the sources and methods of selection of participants. Describe methods of follow-up	6	Lines 70-79
		(*b*) For matched studies, give matching criteria and number of exposed and unexposed	N/A	
Variables	7	Clearly define all outcomes, exposures, predictors, potential confounders, and effect modifiers. Give diagnostic criteria, if applicable	5-6	Lines 45-67
Data sources/ measurement	8*	For each variable of interest, give sources of data and details of methods of assessment (measurement). Describe comparability of assessment methods if there is more than one group	5-6	Lines 45-67
Bias	9	Describe any efforts to address potential sources of bias	7	Lines 94 - 103
Study size	10	Explain how the study size was arrived at	9	Lines 129 - 141
Quantitative variables	11	Explain how quantitative variables were handled in the analyses. If applicable, describe which groupings were chosen and why	6	Lines 69 - 79
Statistical methods	12	(*a*) Describe all statistical methods, including those used to control for confounding	6-7	Lines 69 - 103
		(*b*) Describe any methods used to examine subgroups and interactions	6	Lines 73 – 76
		(*c*) Explain how missing data were addressed	7	Lines 94 - 103
		(*d*) If applicable, explain how loss to follow-up was addressed	N/A	
		(*e*) Describe any sensitivity analyses	7	Lines 94 - 103
**Results**				
Participants	13*	(a) Report numbers of individuals at each stage	8-9	Lines 113 - 141

For peer review only - http://bmjopen.bmj.com/site/about/guidelines.xhtml

		of study—eg numbers potentially eligible, examined for eligibility, confirmed eligible, included in the study, completing follow-up, and analysed		
		(b) Give reasons for non-participation at each stage	8-9	Lines 113 - 141
		(c) Consider use of a flow diagram		Figure 1
Descriptive data	14*	(a) Give characteristics of study participants (eg demographic, clinical, social) and information on exposures and potential confounders	9	Lines 139 - 149
		(b) Indicate number of participants with missing data for each variable of interest	N/A	No data was missing for participants who completed surveys
		(c) Summarise follow-up time (eg, average and total amount)	9	Lines 138 - 149
Outcome data	15*	Report numbers of outcome events or summary measures over time	9-10	Lines 151 - 161
Main results	16	(*a*) Give unadjusted estimates and, if applicable, confounder-adjusted estimates and their precision (eg, 95% confidence interval). Make clear which confounders were adjusted for and why they were included	10	Lines 163 - 180
		(*b*) Report category boundaries when continuous variables were categorized		Table 2
		(*c*) If relevant, consider translating estimates of relative risk into absolute risk for a meaningful time period	9-10	Lines 151 – 161 (we report absolute prevalence of linkage prior to our multivariable model estimations)
Other analyses	17	Report other analyses done—eg analyses of subgroups and interactions, and sensitivity analyses	10	Lines 177 – 180, Supplementary Tables
**Discussion**				
Key results	18	Summarise key results with reference to study objectives	11	Lines 183-191
Limitations	19	Discuss limitations of the study, taking into account sources of potential bias or imprecision. Discuss both direction and magnitude of any potential bias	13	Lines 240-248
Interpretation	20	Give a cautious overall interpretation of results considering objectives, limitations, multiplicity of analyses, results from similar studies, and other relevant evidence	13	Lines 150-254
Generalisability	21	Discuss the generalisability (external validity) of the study results	13	Lines 248-249
**Other information**				
Funding	22	Give the source of funding and the role of the funders for the present study and, if applicable, for the original study on which the present	1	Funding statement

For peer review only

For peer review only - http://bmjopen.bmj.com/site/about/guidelines.xhtml

	article is based

*Give information separately for exposed and unexposed groups.

Note: An Explanation and Elaboration article discusses each checklist item and gives methodological background and published examples of transparent reporting. The STROBE checklist is best used in conjunction with this article (freely available on the Web sites of PLoS Medicine at http://www.plosmedicine.org/, Annals of Internal Medicine at http://www.annals.org/, and Epidemiology at http://www.epidem.com/). Information on the STROBE Initiative is available at http://www.strobe-statement.org.

For peer review only

For peer review only - http://bmjopen.bmj.com/site/about/guidelines.xhtml

BMJ Open

Linkage to primary care after home-based blood pressure screening in rural Kwazulu-Natal, South Africa: A population-based cohort study

Journal:	*BMJ Open*
Manuscript ID	bmjopen-2018-023369.R1
Article Type:	Research
Date Submitted by the Author:	01-Aug-2018

Complete List of Authors:	Siedner, Mark; Massachusetts General Hospital, Medicine; Harvard Medical School,<br>Baisley, Kathy; London School of Hygiene and Tropical Medicine, Epidemiology and Population Health<br>Orne-Gliemann, Joanna; University of Bordeaux<br>Pillay, Deenan; Africa Health Research Institute; University of College London<br>Koole, Olivier; Africa Health Research Institute<br>Wong, Emily; Massachusetts General Hospital<br>Matthews, Philippa; Africa Health Research Institute<br>Tanser, Frank; School of Nursing and Public Health, University of KwaZulu-Natal ; Africa Health Research Institute<br>Herbst, Kobus; Africa Health Research Institute<br>Barnighausen, Till; Africa Health Research Institute<br>Bachmann, Max; UEA, Population Health and Primary Care

**Primary Subject Heading**:	Cardiovascular medicine
Secondary Subject Heading:	Global health, Health services research
Keywords:	Hypertension < CARDIOLOGY, sub-Saharan Africa, Linkage to care, Population health

SCHOLARONE™
Manuscripts

Linkage to primary care after home-based blood pressure screening in rural Kwazulu-Natal, South Africa: A population-based cohort study

Authors:

Mark J. Siedner*[1,2,3]
Kathy Baisley[3,4]
Joanna Orne-Gliemann[5]
Deenan Pillay[3,6]
Olivier Koole[3,4]
Emily B. Wong[1,2,3]
Philippa Matthews[3]
Frank Tanser[3]
Kobus Herbst[3]
Till Barnighausen[3,6,7,8]
Max Bachmann[9]

*Corresponding Author for Inquiries:
Mark J. Siedner
Massachusetts General Hospital Global Health
125 Nashua Street
Boston, MA 02114 USA
T: +1-617-726-4686; F: +1-617-724-1637
msiedner@mgh.harvard.edu

Affiliations:
[1]Massachusetts General Hospital, Boston, USA
[2]Harvard Medical School, Boston, USA
[3]Africa Health Research Institute, KwaZulu-Natal, SA
[4]London School of Tropical Medicine and Hygiene, London, UK
[5]University of Bordeaux, ISPED, INSERM, Bordeaux Population Health Research Center, UMR 1219, Bordeaux, France
[6]University College London, London, UK
[7]University of Heidelberg, Germany
[8]Harvard School of Public Health, Boston, USA
[9]University of East Anglia, Norwich, UK

Funding:
The Africa Health Research Institute receives funding from the United Kingdom Wellcome Trust grant 082384/Z/07/Z. MJS receives funding from the National Institutes of Health (K23 MH099916, P30 30AI060354). EBW receives funding from the National Institutes of Health (K08 AI118538). The population cohort is partly funded by the South African Department of Science and Technology through the SA-MRC hosted South African Population Research Infrastructure Network.

Conflicts of Interest: All authors report no conflicts of interest

For peer review only

ABSTRACT

Objectives: The expanding burden of non-communicable diseases (NCDs) globally will require novel public health strategies. Community-based screening has been promoted to augment efficiency of diagnostic services, but few data are available on the downstream impact of such programs. We sought to assess the impact of a home-based blood pressure screening program on linkage to hypertension care in rural South Africa.

Setting: We conducted home-based blood pressure screening in a population cohort in rural KwaZulu-Natal, using the WHO STEPS protocol.

Participants: Individuals meeting criteria for raised blood pressure (≥140 systolic or ≥90 diastolic averaged over two readings) were referred to local health clinics, and included in this analysis. We defined linkage to care based on self-report of presentation to clinic for hypertension during the next two years of cohort observation. We estimated the population proportion of successful linkage to care with inverse probability sampling weights, and fit multivariable logistic regression models to identify predictors of linkage following a positive hypertension screen.

Results: Of 11,694 individuals screened, 14.6% (n=1,706) were newly diagnosed with elevated pressure. 26.9% (95%CI 24.5-29.4%) of those sought hypertension care in the following two years, and 38.1% (95%CI 35.6-40.7%) did so within five years. Women (aOR 2.41, 95%CI 1.68–3.45), those of older age (aOR 11.49, 95%CI 5.87–22.46, for 45-59 years versus <30), and those unemployed (aOR 1.71, 95%CI 1.10–2.65) were more likely to have linked to care.

Conclusions: Linkage to care after home-based identification of elevated blood pressure was rare in rural South Africa, particularly among younger individuals, men, and the employed. Improved understanding of barriers and facilitators to NCD care is needed to improve the effectiveness of blood pressure screening in the region.

Key Words:
Non-communicable diseases, community health, hypertension, South Africa, linkage to care

Strengths and Limitations of this Study

- Applies a longitudinal population cohort study design with a large sample size to assess linkage to hypertension care after a home-based screening for elevated blood pressure
- Assesses a population in rural sub-Saharan Africa who are noted to have high prevalence of hypertension but with little corresponding data about linkage to care after diagnosis
- Identifies low rates of linkage to care after home-based blood pressure screening in this population, and key factors associated with poor linkage including male sex, younger age, and being employed
- Limitations include low rates of participation in the home-based screening program and incomplete follow-up, as well as self-reported linkage to care as an outcome definition

For peer review only

BACKGROUND

Over two in three deaths worldwide are attributed to non-communicable diseases (NCDs).[1] Although precise measurement of cause-specific mortality in much of the developing world remains a challenge, some estimates suggest that the majority of NCD deaths now occur in low and middle-income countries.[2] In South Africa, for example, the World Health Organization estimates that half of deaths are due to NCDs, and approximately 25% of the population will suffer a premature death due to them.[3]

Consequently, responding to the NCD epidemic in low and middle-income countries is both a major challenge and stated priority of the public health community.[4] NCD morbidity and mortality can be substantially reduced through effective primary and secondary prevention measures targeting risk factors such as smoking, high blood pressure, diabetes, diet and physical activity.[5] Hypertension, which can be controlled through cost-effective lifestyle and pharmacotherapy interventions, is estimated to account for over 50% of the population attributable fraction of stroke in the African region.[6] Yet, in South Africa, national population surveys have estimated that over one quarter of South Africans adults have raised blood pressure, but only approximately one in three of them has received treatment.[7]

The South African Department of Health has outlined strategic NCD goals, which highlight the role for prevention of NCDs and the importance of a community-based focus.[8] One specific strategy includes integrating HIV and NCD screening programs and broadening access to diagnostic and treatment in the community and rural areas. Community-based NCD screening through health fairs and use of community health workers has gained traction recently as a means to efficiently screen large populations of individuals for multiple co-morbidities.[9 10] Whether such endeavors lead to successful linking of individuals to appropriate NCD care is not well established, and is an important question for the field.

In 2010, we conducted a home-based assessment of blood pressure in approximately 12,000 people in a demographic health surveillance (DHS) site in KwaZulu Natal. We referred individuals with raised blood

For peer review only

pressure and not already receiving hypertension treatment to local government clinics for repeat measurement and ongoing hypertension care. We assessed linkage to care during future years of the home-based DHS survey. Our primary aims were to determine the probability of clinical engagement within two years after home-based screening and referral, and to identify predictors of failure to link to care. Our over-arching aim was to inform public health programmers on the feasibility of community-based blood pressure screening as an entry point into NCD care in this setting.

METHODS

Study design, setting, and participants

The African Health Research Institute (AHRI) (formerly the Africa Centre for Health and Population Studies) is a Wellcome Trust funded research institute in South Africa. Since 2000, they have conducted a population cohort study of all adults in a catchment area of 438 km^2 in rural uMkanyakude District, northern KwaZulu-Natal, covering a total population of approximately 100,000 individuals.[11] Households are surveyed 2–3 times per year, to collect information on birth, deaths and migration patterns for all household members, including non-residents. Since 2003, resident household members ≥15 years have been invited to participate in an annual home-based individual survey, which collects data on sociodemographics and general health information.

Blood pressure screening and referral methods

In 2010, all individuals who participated in the home-based survey were also offered a physical examination to determine weight, height and blood pressure, using the WHO STEPS protocol.[12] Blood pressure was measured using Omron automated blood pressure monitors (Omron Global, Kyoto Japan). Blood pressure was measured after 15 minutes of resting in a seated position. We collected three measurements, each five minutes apart, with the mean of the last two measurements used to identify those with elevated blood pressure. A positive hypertension screen was defined as a mean systolic blood pressure ≥140 mmHg and/or diastolic blood pressure ≥90 mmHg.[13] All individuals were given a copy of

For peer review only

their blood pressure results, along with body mass index, on a health results card. Those with elevated blood pressure were counselled by study staff to seek care at their preferred local public health clinic with a copy of their screening results.

Outcome assessment

For our primary outcome of interest, we defined successful linkage to care for hypertension as self-reported linkage within two years of a positive home-based hypertension screen. To assess this outcome, we used data from the two subsequent, annual home-based health surveys in 2011 and 2012. All participants who took part in the initial blood pressure screening survey were members of the demographic health and surveillance site study population, and are seen annually at home for data collection. In each annual health survey, respondents were asked if: 1) they have been diagnosed with hypertension in the past 12 months; 2) if they have ever received hypertension treatment; and 3) if they are currently being treated for hypertension. We defined successful linkage to care by a positive response to any of these three questions in either 2011 or 2012. As secondary outcomes of interest, we also examined 1) linkage to hypertension care within five years (as opposed to two), defined as a positive response to any of the same three questions in the annual health surveys during 2011-2015 and 2) confirmation of hypertension care seven years after the screening, as evidenced by clinical records from all public health clinics in the catchment area in 2017, the first year linked clinical data was linked to the population cohort database.

Statistical methods

We included in this analysis individuals who had a positive hypertension screen in the home-based 2010 survey, and who reported no previous diagnosis of hypertension or hypertension treatment. We first summarized sociodemographic characteristics of eligible adults who had blood pressure measurements in the 2010 survey. We then estimated population-level prevalence of linkage to hypertension care in the two years after the screening program, both overall and stratified by sex and age, with the use of inverse

For peer review only

probability weights (IPWs) of the probability of participating in the hypertension screening. We used IPWs to make the results generalizable to the entire 2010 sample. To calculate the weights, we fit a logistic regression model with completion of blood pressure screening in 2010 as the outcome of interest and included age strata, sex, education level and place of residence (urban, peri-urban, or rural) as predictors, based on information routinely collected in the household-level survey.

We then fit logistic regression models to estimate odds ratios (OR) and 95% confidence intervals (CI) for factors independently associated with linkage to hypertension care within two years of a positive home-based hypertension screen. Potential determinants of linkage were examined at three levels: sociodemographic factors (age, sex, educational attainment, employment status and socioeconomic status, which was estimated using principal components analysis of household asset ownership following the method of Filmer and Pritchett[14]); geographical factors (distance from clinic, urban versus rural residency); and clinical factors (body mass index, elevated blood pressure severity [defined using hypertension stages as a) stage I: systolic 140-160 and diastolic 80-100; b) stage II: systolic 160-<180 or diastolic 100-<120; or c) hypertensive urgency: systolic ≥180 or diastolic ≥120], self-report of diabetes, self-report of tuberculosis)[15]. Self-reported data on HIV diagnosis was not collected in the survey, so could not be included in the analysis. Sociodemographic and clinical factors whose age- and sex-adjusted association with linkage was significant at p<0.10 were included in a final adjusted multivariable model. Distance from the nearest clinic was analyzed as a continuous covariate. In order to allow for non-linear relationships between distance and linkage to care, we used fractional polynomial functions.[16]

We tested the robustness of our findings using several sensitivity analyses. First, we changed our outcome from self-reported linkage to care in 2011 or 2012 to 1) self-reported linkage to care at any time between 2011-2015, and 2) confirmation of a clinic appointment for hypertension in 2017 at any of the 11 local public sector clinics, among those who remained a resident in the catchment area. Next, we compared characteristics of eligible individuals who did and did not complete blood pressure screening in 2010. Next,

For peer review only - http://bmjopen.bmj.com/site/about/guidelines.xhtml

we compared characteristics of those who participated in a subsequent health survey and those who did not, either because of refusal, out-migration, or death. Finally, we conducted sensitivity analyses in which we: 1) used IPWs of screening in the models; and 2) added a covariate to indicate the number of individual health surveys participated in during 2011-2015. Data were entered and verified in an SQL database, and were analyzed using Stata 14 (StataCorp, College Station, TX).

Human Studies Considerations

Ethical approval for the demographic surveillance study and analyses of these data was granted by the Biomedical Research Ethics Committee of the University of KwaZulu-Natal, South Africa. Separate informed consent was given for the demographics survey, the blood pressure screening, and the clinic records abstraction.

Patient and Public Involvement

Patients were not involved in the design of this study. This analysis was designed by study investigators at the Africa Health Research Institute intent on leveraging prior home-based screening protocols to inform and optimize future community-based research, and particularly to improve the public health impact of such activities. The results of this study were presented to the South African Department of Health Non-Communicable Diseases Unit and will be disseminated to the community during the monthly scheduled Africa Health Research Institute community road shows.

RESULTS

Survey participants

A total of 37,693 potentially eligible adults were in the sampling frame. Of these, approximately one quarter (8,589, 22.8%) were not available due to out-migration, death or inability to consent and another 2,920 (7.7%) could not be contacted (Figure 1). Of the remaining 26,184 individuals who were contacted and eligible for the home-based DHS survey in 2010, 11,814 (45.1%) consented to participate in the

For peer review only

general health survey and 11,694 (44.7%) had valid blood pressure measurements. Women, older individuals, and those of lower socioeconomic position and education were more likely to participate in the survey (Supplementary Table 1).

The majority of participants with a blood pressure measurement were women (n=8,241, 70.5%, Table 1). Median age was 25 years (interquartile range [IQR] 18–47 years) for men and 38 years (IQR 23–55 years) for women. The majority of participants (n=7,464, 63.8%) resided in a rural setting, and less than one quarter (n=2,642, 22.6%) lived within 1.5 kilometers of the nearest clinic. Few participants (n=1,779, 15.2%) were currently employed.

Screening for hypertension

Approximately one quarter (n=3,074, 26.2%) of participants were found to have elevated pressure during the home-based blood pressure screening, of whom 1,368 (44.5%) reported having been previously diagnosed or currently on treatment. Of those who had been previously diagnosed or in hypertension care, 1,169 (85.5%) were currently on hypertension treatment. Participants who were not previously aware of their condition were significantly younger, and more likely to be men, married, employed, have a higher level of education, and be living in peri-urban areas than those who had been previously diagnosed or on treatment (Table 1). However, there was no evidence of a difference between the two groups in the distance from their nearest clinic.

Analytic Sample

A total of 1,199 individuals (70.3%) who were not previously aware of having elevated blood pressure participated in a second general health survey within two years of being screened (that is, in 2011 or 2012), and were included in the primary analysis of factors associated with linkage to hypertension care. Compared with the 507 individuals who did not participate in 2011 or 2012, those who participated in 2011 or 2012 were older (median (IQR) age = 50 (38–66) years, vs 43 (29–58) years), more likely to be

For peer review only

women, unmarried, have lower levels of education, be unemployed, and have a higher BMI (Supplementary Table 2). There was no difference in participation rates by distance from the nearest clinic. When we expanded the observation period to include surveys from 2011-2015, a total of 1,421 (83.3%) participated in at least one home-based annual general health survey. Of the 285 (16.7%) individuals who did not participate in any health survey after 2010, 81 out-migrated and 36 died before the 2011 survey (Figure 1). The remaining 168 were eligible for at least one subsequent survey but refused participation.

Linkage to subsequent hypertension care

The crude and, IPW-adjusted population prevalence of linkage to hypertension care within two years of the blood pressure screen was 28.6% (95%CI=26.1-31.2%), and 26.9% (95%CI=24.5-29.4%), respectively. Of the 343 total new linkages reported, 218 (64%) and 135 (36%) were reported in 2011 and 2012, respectively. Of the 218 linkages reported in 2011, the majority (191, 88%) reported initiating anti-hypertensive therapy. Using IPW-adjusted estimates, we found that women were more likely than men to link to care, and older individuals were more likely than younger individuals (Figure 2), such that we estimate that 44.9% (95%CI=39.4-50.5%) of women ≥60 years presented to care for hypertension in the next two years, versus only 3.0% (95%CI=1.1-7.7%) of men under 45. When we extended our surveillance period out to 2015, we estimate that 38.1% (95%CI=35.6-40.7%) of individuals reported linking to hypertension care within 5 years. Finally, we found that only 16.6% (95%CI=14.6-18.9%) of individuals who remained a resident in 2016 and who screened positive for elevated blood pressure in 2010 completed a clinic appointment for hypertension at one of the public health clinics in the catchment area in 2016.

Factors associated with presentation to hypertension care within two years

In models adjusted for socioeconomic, geographic, and clinical factors, we found strong evidence that women (OR=2.76, 95%CI=1.97 – 3.88, p<0.001) and those of older age (OR=12.89, 95%CI 6.62 – 25.11, p<0.0001, comparing those 45-59 years versus those <30) were more likely to present to hypertension care within two years of home-based diagnosis (Table 2). In adjusted analysis, the association with age and sex

For peer review only

remained statistically significant, and there was no evidence that the effect of age on linkage to care differed between men and women (p-value for interaction=0.20, Figure 2). There was evidence that those who were unemployed were more likely to link to care (adjusted (a)OR 2.09, 95%CI 1.39 – 3.14). There was an association between distance from clinic and linkage to hypertension care such that odds of presentation increased as distance to the clinic increased (aOR for linear trend in linkage with each 1 km increase in distance = 1.12, 95%CI=1.05–1.20, p<0.001). The results of the fractional polynomial models suggested that the linear model adequately described the relationship between presentation to care and distance. After adjusting for sociodemographic and location factors, we also found strong evidence that individuals with the equivalent of Stage II hypertension (aOR=2.20, 95%CI=1.63 – 2.97), and those meeting criteria for hypertensive urgency (aOR=3.07, 95%CI=2.01 – 4.67) had higher odds of linking to care than those with the equivalent of Stage I hypertension. We found similar correlates of presentation to hypertension care (age, sex, distance from clinic, and employment) in sensitivity analyses with weighted models, and in models with a covariate for the number of follow-up surveys completed during 2011-2015 (Supplementary Tables 3-5).

DISCUSSION

We found very low rates of presentation to care after home-based identification of elevated blood pressure in rural KwaZulu Natal. Overall, less than one third of individuals newly identified with elevated blood pressure reported being diagnosed with hypertension or receiving treatment for elevated blood pressure from a clinic within two years, and less than one in five had evidence of visiting a clinic for hypertension care during a 12-month period seven years after the screen. Linkage rates were particularly low for men and young people. Notably, those employed and those closest to clinics also had poorer rates of linkage. These results highlight the important need to consider the determinants of healthcare access for NCDs in rural South Africa, and multi-faceted approaches to improve linkage to care after community-based NCD screening programs.

For peer review only

Linkage with clinical programs after community- and home-based disease screening for chronic disease in sub-Saharan Africa have demonstrated mixed results. Most evidence has come from the HIV field, in which linkage after home-based testing in pilot studies has been highly successful,[17] although lower rates are reported in community settings.[9 18] Studies reporting clinic attendance after hypertension screening have generally shown low rates of linkage to care. For example, in a large (n=6,000) health fair-based screening program in Uganda, 41% of participants with a new positive screen for elevated blood pressure linked to care.[9] A pilot study in Kenya that compared a home-based (n=236) with a community-based healthfair booth approach (n=346) for hypertension and diabetes screening, found equally low rates of linkage to care (30%) with both strategies[19] . A smaller study in Kenya yielded higher linkage rates (74%, n=120) after community group-initiated blood pressure screening.[20] Interpreting these contrasting results must be done with attention to the selection criteria of each. Whereas our procedures were home-based, the larger study from Uganda included self-referring individuals who had attended a health fair, and the Kenyan study operated through a peer microfinance program, in which NCD screening services were paired with agribusiness advice within pre-organized community groups. In the prior report most similar to ours, a large program in Malawi (n=27,305) that provided clinical referrals after home-based testing reported a 59% linkage rate within two weeks of a diagnosis of hypertension, although 30% of participants were already on treatment at the time of referral.[21] Moreover, approximately 50% dropped out of care within 6 months of linkage.

Predictors of presentation to care in our study reinforce much of the literature on health care access and engagement among vulnerable populations in sub-Saharan Africa. Lower engagement by younger individuals and men are well-established phenomena; and a public health challenge for the region.[22 23] Although it did not reach statistical significance, we also found evidence that those with greater social support, as evidenced by having a cohabitating partner, had a 35% increased odds of presenting to care. An unexpected finding was that those who were unemployed and those further from clinic were more likely to link to care. This finding contrasts with much of the data from the region on how distance from

For peer review only - http://bmjopen.bmj.com/site/about/guidelines.xhtml

health services impacts linkage to and retention in care.[24-26] We hypothesize that these results illustrate competing demands between obligations to work and to access healthcare. Notably, a similar phenomenon was found in the Malawi home-based NCD screening study, in which rural participants had more than twice the odds of linkage to NCD care than their urban counterparts, and the most common reason stated for failure to link to care in urban areas was being too busy to attend clinic; reported in 34% of those not linked to care.[21] Employment is higher in male than in female South African blacks [27], and so may also contribute to the gender difference in linkage.

Our data do offer multiple potential strategies to improve linkage to care after home-based NCD screening. For example, a notable distinction between many community-based HIV and NCD diagnostic programs is the degree of counseling and referral services provided after diagnosis. Hypertension referral services in our program and many others in the region are often limited to distribution of results and referral forms. The potential beneficial effects of more comprehensive health and lifestyle counseling on the success of linkage after an elevated blood pressure screen should be actively explored in future work. For example, decades of standardizing in-depth HIV counseling services, and additional facilitated linkage strategies have significantly improved rates of linkage after a new HIV diagnosis.[28] Pilot studies of enhanced referral after community based NCD diagnoses have also shown promise in vulnerable populations in the United States,[29 30] and warrant investigation on a larger scale elsewhere. Similarly, our finding and that of others that hypertension linkage was less common in those employed presents a potential opportunity to consider expanded clinic service hours and/or community based management to improve NCD care in the region. Endeavors, such as the Centralised Chronic Medication Dispensing and Distribution recently launched by the KwaZulu Natal Department of Health, seek to overcome such barriers by delivering medicines to peoples' homes and workplaces, or setting up community-based medicine pickup points. Evaluations of the efficacy and sustainability of such programs will be of high importance to the field. Finally, our results, and particularly the high prevalence of elevated blood pressure and low rates of care sinking after notification of such, highlight the critical importance of risk factor modification as part of

For peer review only

public health strategies. Such programs, including smoking cessation, healthy diet, and exercise promotion, which are recommended by South African Department of Health hypertension control policies, have potential to significantly impact health, and must also remain a cornerstone of population hypertension control rograms.[31 32]

Our study is strengthened by a large sample size and the use of a home-based testing paradigm. The primary limitation to our analysis is the relatively low response rate in the initial hypertension screen and participation in subsequent surveys. We accounted for this limitation by comparing characteristics between participants and non-participants, and by using inverse probability weighting techniques to make population level inferences. We hypothesize that the low participation in this instance was due to the placement of the screening activity within a routine annual survey, as opposed to a stand-alone health promotion. Nonetheless, it should be noted that a similar response rate for health interventions would also pose a challenge to their use for population-wide screening programs. Our study is also limited by a use of self-report to detect linkage to clinical care over the first two years of observation and clinic records seven years later, which may lead to misestimation of outcomes. For example, participants who presented to clinic and had a normal blood pressure might not be detected by self-report of a new diagnosis of hypertension or with use of clinical records to seven years after the initial screen. Our study also did not investigate if and how supply-side considerations, such as staffing, wait times, and drug availability might have contributed to the low rates of linkage. Although hypertension care and treatment is provided free of charge in South Africa in health clinics in the public sector, these factors have been demonstrated to affect engagement in care elsewhere in the country.[33 34] Finally, our results should be considered in the context of the low-resource, rural sub-Saharan Africa study stetting, but are unlikely to generalize more broadly to urban or higher resource regions.

In summary, we found very low rates of linkage to care after a population-level, home-based hypertension screen in rural KwaZulu Natal. Strategies focused on increased demand generation, particularly for

For peer review only

younger individuals and men, augmented referral and linkage programs, and efforts to enhance the convenience of service delivery, particularly to employed people, should be evaluated to improve NCD care access after community based testing in the region.

For peer review only

Author Contributions:

MJS: Conceived of the project, wrote the first draft, and contributed to the data analysis.

KB: Led the data analysis, contributed to manuscript production.

JOB: Helped conceive of the project and contributed to manuscript production.

DP: Contributed to data collection, project conception, and contributed to manuscript production.

OK: Contributed to data collection, project conception, and contributed to manuscript production.

EW: Contributed to data collection and project conception.

PM: Contributed to data collection, project conception, and contributed to manuscript production.

FT: Contributed to data collection and project conception.

KH: Contributed to data collection and project conception.

TB: Contributed to data collection, project conception, and contributed to manuscript production.

MB: Contributed to project conception, data analysis, and contributed to manuscript production.

All authors reviewed the manuscript for editorial contributions and approved of the final version.

Data Sharing:

Data for this analysis is available by emailing Mark Siedner (msiedner@mgh.harvard.edu) and Kobus Herbst (kherbst@ahri.org)

For peer review only

REFERENCES

1. Lozano R, Naghavi M, Foreman K, et al. Global and regional mortality from 235 causes of death for 20 age groups in 1990 and 2010: a systematic analysis for the Global Burden of Disease Study 2010. *Lancet* 2012;380(9859):2095-128. doi: 10.1016/S0140-6736(12)61728-0
2. Alwan A, Maclean DR, Riley LM, et al. Monitoring and surveillance of chronic non-communicable diseases: progress and capacity in high-burden countries. *Lancet* 2010;376(9755):1861-8. doi: 10.1016/S0140-6736(10)61853-3
3. Non-communicable Diseases Progress Monitor. World Health Organization. 2017. Avaialble at: https://ncdalliance.org/sites/default/files/resource_files/WHOProgressMonitor2017.pdf. Accessed 21st November 2017.
4. Global Action Plan for the Prevention and Control of Non-Communicable Diseases 2013-2020. World Health Organization. Available at: http://apps.who.int/iris/bitstream/10665/94384/1/9789241506236_eng.pdf. Accessed 21st November, 2017.
5. Yusuf S, Hawken S, Ounpuu S, et al. Effect of potentially modifiable risk factors associated with myocardial infarction in 52 countries (the INTERHEART study): case-control study. *Lancet* 2004;364(9438):937-52. doi: 10.1016/S0140-6736(04)17018-9 [published Online First: 2004/09/15]
6. O'Donnell MJ, Chin SL, Rangarajan S, et al. Global and regional effects of potentially modifiable risk factors associated with acute stroke in 32 countries (INTERSTROKE): a case-control study. *Lancet* 2016;388(10046):761-75. doi: 10.1016/S0140-6736(16)30506-2 [published Online First: 2016/07/20]
7. Health Systems Trust. South African Health Review, 2016.
8. Strategic Plan for the Prevention and Control of Non-Communicable Diseases 2013-17. South African Ministry of Health. Available at: http://www.hsrc.ac.za/uploads/pageContent/3893/NCDs STRAT PLAN_CONTENT 8 april proof.pdf Accessed 21st November 2017.
9. Chamie G, Kwarisiima D, Clark TD, et al. Leveraging rapid community-based HIV testing campaigns for non-communicable diseases in rural Uganda. *PLoS One* 2012;7(8):e43400. doi: 10.1371/journal.pone.0043400 [published Online First: 2012/08/24]
10. Gaziano TA, Abrahams-Gessel S, Denman CA, et al. An assessment of community health workers' ability to screen for cardiovascular disease risk with a simple, non-invasive risk assessment instrument in Bangladesh, Guatemala, Mexico, and South Africa: an observational study. *The Lancet Global health* 2015;3(9):e556-63. doi: 10.1016/S2214-109X(15)00143-6 [published Online First: 2015/07/19]
11. Tanser F, Hosegood V, Barnighausen T, et al. Cohort Profile: Africa Centre Demographic Information System (ACDIS) and population-based HIV survey. *International journal of epidemiology* 2008;37(5):956-62. doi: 10.1093/ije/dym211 [published Online First: 2007/11/14]
12. World Health Organization. Noncommunicable diseases and mental health cluster. WHO STEPS Surveillance Manual: The WHO STEPwise Approach to Chronic Disease Risk Factor Suveillance. Geneva. 2005
13. Malaza A, Mossong J, Barnighausen T, et al. Hypertension and obesity in adults living in a high HIV prevalence rural area in South Africa. *PLoS One* 2012;7(10):e47761. doi: 10.1371/journal.pone.0047761
14. Filmer D, Pritchett LH. Estimating wealth effects without expenditure data--or tears: an application to educational enrollments in states of India. *Demography* 2001;38(1):115-32. [published Online First: 2001/03/03]
15. Glanz K, Rimer BK, Viswanath K. Health behavior and health education: Theory, Research and Practice. 4th ed. San Francisco, CA: Jossey-Bass 2008.
16. Royston P, Ambler G, Sauerbrei W. The use of fractional polynomials to model continuous risk variables in epidemiology. *International journal of epidemiology* 1999;28(5):964-74. [published Online First: 1999/12/22]
17. van Rooyen H, Barnabas RV, Baeten JM, et al. High HIV testing uptake and linkage to care in a novel program of home-based HIV counseling and testing with facilitated referral in KwaZulu-Natal, South

For peer review only

Africa. *J Acquir Immune Defic Syndr* 2013;64(1):e1-8. doi: 10.1097/QAI.0b013e31829b567d [published Online First: 2013/05/30]

18. Iwuji CC, Orne-Gliemann J, Larmarange J, et al. Universal test and treat and the HIV epidemic in rural South Africa: a phase 4, open-label, community cluster randomised trial. *Lancet HIV* 2017 doi: 10.1016/S2352-3018(17)30205-9 [published Online First: 2017/12/05]
19. Pastakia SD, Ali SM, Kamano JH, et al. Screening for diabetes and hypertension in a rural low income setting in western Kenya utilizing home-based and community-based strategies. *Globalization and health* 2013;9:21. doi: 10.1186/1744-8603-9-21 [published Online First: 2013/05/18]
20. Pastakia SD, Manyara SM, Vedanthan R, et al. Impact of Bridging Income Generation with Group Integrated Care (BIGPIC) on Hypertension and Diabetes in Rural Western Kenya. *J Gen Intern Med* 2017;32(5):540-48. doi: 10.1007/s11606-016-3918-5 [published Online First: 2016/12/07]
21. Musicha C, Crampin AC, Kayuni N, et al. Accessing clinical services and retention in care following screening for hypertension and diabetes among Malawian adults: an urban/rural comparison. *Journal of hypertension* 2016;34(11):2172-9. doi: 10.1097/HJH.0000000000001070 [published Online First: 2016/08/24]
22. Kotwani P, Balzer L, Kwarisiima D, et al. Evaluating linkage to care for hypertension after community-based screening in rural Uganda. *Trop Med Int Health* 2014;19(4):459-68. doi: 10.1111/tmi.12273 [published Online First: 2014/02/06]
23. Zack RM, Irema K, Kazonda P, et al. Determinants of high blood pressure and barriers to diagnosis and treatment in Dar es Salaam, Tanzania. *Journal of hypertension* 2016;34(12):2353-64. doi: 10.1097/HJH.0000000000001117 [published Online First: 2016/11/03]
24. Lankowski AJ, Siedner MJ, Bangsberg DR, et al. Impact of geographic and transportation-related barriers on HIV outcomes in sub-Saharan Africa: a systematic review. *AIDS Behav* 2014;18(7):1199-223. doi: 10.1007/s10461-014-0729-8
25. Siedner MJ, Lankowski A, Tsai AC, et al. GPS-measured distance to clinic, but not self-reported transportation factors, are associated with missed HIV clinic visits in rural Uganda. *AIDS* 2013;27(9):1503-8. doi: 10.1097/QAD.0b013e32835fd873
26. Tanser F, Gijsbertsen B, Herbst K. Modelling and understanding primary health care accessibility and utilization in rural South Africa: an exploration using a geographical information system. *Social science & medicine* 2006;63(3):691-705. doi: 10.1016/j.socscimed.2006.01.015 [published Online First: 2006/04/01]
27. Statistics South Africa. Gender statistics in South Africa, 2011. http://www.statssa.gov.za/publications/Report-03-10-05/Report-03-10-052011.pdf (accessed 27 February 2017). .
28. Sharma M, Ying R, Tarr G, et al. Systematic review and meta-analysis of community and facility-based HIV testing to address linkage to care gaps in sub-Saharan Africa. *Nature* 2015;528(7580):S77-85. doi: 10.1038/nature16044 [published Online First: 2015/12/04]
29. Krieger J, Collier C, Song L, et al. Linking community-based blood pressure measurement to clinical care: a randomized controlled trial of outreach and tracking by community health workers. *Am J Public Health* 1999;89(6):856-61. [published Online First: 1999/06/08]
30. Baig AA, Mangione CM, Sorrell-Thompson AL, et al. A randomized community-based intervention trial comparing faith community nurse referrals to telephone-assisted physician appointments for health fair participants with elevated blood pressure. *J Gen Intern Med* 2010;25(7):701-9. doi: 10.1007/s11606-010-1326-9 [published Online First: 2010/03/30]
31. Rose G. Sick individuals and sick populations. *International journal of epidemiology* 2001;30(3):427-32; discussion 33-4. [published Online First: 2001/06/21]
32. South African Health Review. Health Systems Trust. Durban, South Africa. 2016. Available at: https://www.health-e.org.za/wp-content/uploads/2016/05/South-African-Health-Review-2016.pdf; Accessed 8th January 2018.
33. Wollum A, Gabert R, McNellan CR, et al. Identifying gaps in the continuum of care for cardiovascular disease and diabetes in two communities in South Africa: Baseline findings from the HealthRise

For peer review only - http://bmjopen.bmj.com/site/about/guidelines.xhtml

project. *PLoS One* 2018;13(3):e0192603. doi: 10.1371/journal.pone.0192603 [published Online First: 2018/03/15]

34. Ameh S, Klipstein-Grobusch K, D'Ambruoso L, et al. Quality of integrated chronic disease care in rural South Africa: user and provider perspectives. *Health policy and planning* 2017;32(2):257-66. doi: 10.1093/heapol/czw118 [published Online First: 2017/02/17]

For peer review only

Table 1. Participants with blood pressure measured in in 2010 survey, those with hypertension[1] and stratified by whether or not previously diagnosed or on treatment

	All participants	N with hypertension[1]	Previously diagnosed[2]	Not previously diagnosed[3]
	N=11,694	N=3,074 (26.3%)	N=1,368 (44.5%)	N=1,706 (55.5%)
**Median (IQR) age (years)**	34 (21–53)	55 (43–68)	60 (51–71)	49 (35–63)
**Age group**				P<0.001[4]
<30	5,107 (43.7%)	354 (11.5%)	21 (1.5 %)	333 (19.5%)
35-44	2,191 (18.7%)	484 (15.7%)	118 (8.6 %)	366 (21.5%)
45-59	2,265 (19.4%)	992 (32.3%)	507 (37.1%)	485 (28.4%)
60+	2,131 (18.2%)	1,244 (40.5%)	722 (52.8%)	522 (30.6%)
**Sex**				P<0.001
Male	3,453 (29.5%)	720 (23.4%)	178 (13.0%)	542 (31.8%)
Female	8,241 (70.5%)	2,354 (76.6%)	1190 (87.0%)	1164 (68.2%)
**Education**				P<0.001
None	2,389 (20.5%)	1,032 (33.6%)	550 (40.2%)	482 (28.3%)
Less than complete secondary	6,244 (53.5%)	1,463 (47.6%)	662 (48.4%)	801 (47.0%)
Complete secondary/above	3,040 (26.0%)	576 (18.8%)	155 (11.3%)	421 (24.7%)
Missing	*21*	*3*	*1*	*2*
**Marital status**				P<0.001
Single (never married)	3,462 (29.8%)	518 (16.9%)	174 (12.7%)	344 (20.2%)
Married/informal union	6,556 (56.3%)	1,696 (55.2%)	688 (50.3%)	1008 (59.2%)
Widowed/separated/divorced	1,618 (13.9%)	857 (27.9%)	506 (37.0%)	351 (20.6%)
Missing	*58*	*3*	*0*	*3*
**Employed**				P<0.001
Yes	1,779 (15.3%)	437 (14.2%)	140 (10.2%)	297 (17.4%)
No	9,828 (84.7%)	2,634 (85.8%)	1,228 (89.8%)	1406 (82.6%)
Missing	*87*	*3*	*0*	*3*
**Residence**				P<0.001
Urban	617 (5.3 %)	119 (3.9 %)	55 (4.0 %)	64 (3.8 %)
Peri-urban	3,604 (30.8%)	904 (29.4%)	347 (25.4%)	557 (32.7%)
Rural	7,464 (63.9%)	2,050 (66.7%)	966 (70.6%)	1084 (63.6%)
Missing	*9*	*1*	*0*	*1*
**SES tertile**				P=0.67
Low	4193 (36.4%)	1173 (38.6%)	525 (38.7%)	648 (38.5%)
Middle	3818 (33.1%)	947 (31.2%)	412 (30.4%)	535 (31.8%)
High	3522 (30.5%)	918 (30.2%)	418 (30.8%)	500 (29.7%)
Missing	*161*	*36*	*13*	*23*
**Self-report of diabetes**[5]				P<0.001
No	11300 (96.6%)	2867 (93.3%)	1176 (86.0%)	1691 (99.1%)
Yes	394 (3.4 %)	207 (6.7 %)	192 (14.0%)	15 (0.9 %)
**Nearest clinic (km)** [6]				P=0.84
0- <1.5	2642 (22.6%)	676 (22.0%)	292 (21.3%)	384 (22.5%)
1.5-2.5	2879 (24.6%)	710 (23.1%)	314 (23.0%)	396 (23.2%)
>2.5-3.9	2975 (25.5%)	809 (26.3%)	368 (26.9%)	441 (25.9%)
>3.9	3189 (27.3%)	878 (28.6%)	394 (28.8%)	484 (28.4%)
Missing	*9*	*1*	*0*	*1*

[1]Hypertension defined as systolic BP ≥ 140mmHg or diastolic BP ≥90 mmHg, in an average of 2 readings. [2]Report having been previously diagnosed or on treatment for hypertension in 2010 survey. [3]Report no previous diagnosis or treatment for hypertension in 2010 survey. [4]P-value from Chi-squared test comparing characteristics of those previously diagnosed/treatment and those with no previous diagnosis/treatment. [5]Report having been diagnosed with or on treatment for diabetes in 2010 survey. [6]Quartiles based on distribution in all individuals who were eligible for 2010 survey.

For peer review only

Table 2. Factors[1] associated with linkage to hypertension care within 2 years after home-based diagnosis of hypertension[2] in 2010, among individuals who were previously undiagnosed and participated in 2011 or 2012 (N=1199) (unweighted analysis)

	Linked to care/N (%)	Crude OR (95% CI)	Age- & sex-adjusted OR (95% CI)	Adjusted OR (95% CI)[3]
**Sociodemographic factors**				
**Age group**		P<0.001	P<0.001	P<0.001
<30	10 / 193 (5.2 %)	1	1	1
35-44	40 / 242 (16.5%)	3.62 (1.76 -7.45)	3.08 (1.49 -6.36)	3.32 (1.60 -6.89)
45-59	126 / 360 (35.0%)	9.85 (5.03 -19.30)	8.39 (4.26 -16.51)	9.01 (4.57 -17.79)
60+	167 / 404 (41.3%)	12.89 (6.62 -25.11)	11.61 (5.94 -22.69)	11.49 (5.87 -22.46)
**Sex**		P<0.001	P<0.001	P<0.001
Male	47 / 308 (15.3%)	1	1	1
Female	296 / 891 (33.2%)	2.76 (1.97 -3.88)	2.50 (1.75 -3.57)	2.41 (1.68 -3.45)
**Marital status**		P<0.001	P=0.15	P=0.14
Single (never married)	55 / 250 (22.0%)	1	1	1
Married/informal union	178 / 666 (26.7%)	1.29 (0.92 -1.83)	1.33 (0.91 -1.95)	1.35 (0.92 -1.98)
Widow/sep/divorced	110 / 283 (38.9%)	2.25 (1.54 -3.31)	0.98 (0.64 -1.50)	0.99 (0.65 -1.51)
**Education**		P<0.001	P=0.83	P=0.77
None	132 / 363 (36.4%)	1	1	1
Less than complete secondary	160 / 581 (27.5%)	0.67 (0.50 -0.88)	1.10 (0.81 -1.49)	1.09 (0.80 -1.49)
Complete secondary/above	51 / 255 (20.0%)	0.44 (0.30 -0.64)	1.09 (0.71 -1.67)	1.15 (0.75 -1.78)
**Employed**		P<0.001	P=0.02	P=0.02
Yes	31 / 178 (17.4%)	1	1	1
No	312 / 1021 (30.6%)	2.09 (1.39 -3.14)	1.71 (1.10 -2.65)	1.71 (1.10 -2.65)
**SES tertile**		P=0.307	P=0.31	P=0.21
Low	125 / 459 (27.2%)	1	1	1
Middle	99 / 364 (27.2%)	1.00 (0.73 -1.36)	1.09 (0.79 -1.52)	1.12 (0.81 -1.56)
High	115 / 364 (31.6%)	1.23 (0.91 -1.67)	1.28 (0.93 -1.77)	1.34 (0.97 -1.85)
**Location factors**				
**Residence**		P=0.04	P=0.35	P=0.55
Urban	10 / 36 (27.8%)	1	1	1
Peri-urban	95 / 398 (23.9%)	0.82 (0.38 -1.75)	0.75 (0.33 -1.69)	0.67 (0.29 -1.53)
Rural	238 / 765 (31.1%)	1.17 (0.56 -2.47)	0.92 (0.41 -2.05)	0.63 (0.27 -1.45)
**Nearest clinic**[4]		P<0.001	P=0.001	P=0.001
Per each km of distance		1.15 (1.08 -1.23)[5]	1.13 (1.05 -1.21)[5]	1.12 (1.05 -1.20)[5]
0- <1.5	56 / 263 (21.3%)			
1.5-2.5	71 / 269 (26.4%)			
>2.5-3.9	93 / 310 (30.0%)			
>3.9	123 / 357 (34.5%)			
**Clinical factors**				
**BMI category**		P<0.001	P=0.07	P=0.13
<25 kg/m^2	70 / 344 (20.3%)	1	1	1
25 – <30 kg/m^2	62 / 229 (27.1%)	1.45 (0.98 -2.15)	1.08 (0.71 -1.64)	1.17 (0.76 -1.81)
≥30 kg/m^2	110 / 301 (36.5%)	2.25 (1.59 -3.21)	1.52 (1.03 -2.24)	1.51 (1.00 -2.26)
**Hypertension stage**[6]		P<0.001	P<0.001	P<0.001
Stage I	142 / 730 (19.5%)	1	1	1
Stage II	134 / 342 (39.2%)	2.67 (2.01 -3.54)	2.22 (1.65 -2.99)	2.20 (1.63 -2.97)
Hypertension urgency	67 / 127 (52.8%)	4.62 (3.12 -6.85)	3.12 (2.06 -4.74)	3.07 (2.01 -4.67)
**Self-report of diabetes**[7]		P=0.19	P=0.44	P=0.47
No	339 / 1191 (28.5%)	1	1	1
Yes	4 / 8 (50.0%)	2.51 (0.62 -10.11)	1.78 (0.41 -7.70)	1.75 (0.38 -8.15)

**Self-report of TB**[7]		P=0.66	P=0.96	P=0.72
No	332 / 1156 (28.7%)	1	1	1
Yes	11 / 43 (25.6%)	0.85 (0.43 -1.71)	1.02 (0.49 -2.13)	1.15 (0.54 -2.46)

[1]All characteristics are based on 2010 survey. [2]Hypertension defined as systolic BP ≥ 140mmHg or diastolic BP ≥90 mmHg, in an average of 2 readings. [3]Sociodemographic factors adjusted for age group, sex, and employment. Location factors adjusted for age group, sex, employment, and distance from nearest clinic as continuous covariate. Clinical factors adjusted for age group, sex, employment, distance from nearest clinic and hypertension stage. [4]Quartiles based on distribution in all individuals who were eligible for 2010 survey. Fit as continuous covariate; n (%) linked in each distance quartile shown for information only. [5]OR for linear trend in linkage with each 1 km increase in distance. [6]Stage I: Systolic BP 140–159 or diastolic BP 90–99; Stage II: Systolic BP 160–179 or diastolic BP 100–119; Hypertension urgency: Systolic BP ≥180 or diastolic BP ≥120. [7]Reports being diagnosed in the past 12m or currently on treatment

For peer review only

Figure 1. Flow diagram of eligible and included participants in a baseline community-based hypertension screen in 2010 and follow-up observation during 2011/2012

For peer review only

For peer review only - http://bmjopen.bmj.com/site/about/guidelines.xhtml

Figure 2. Proportion of individuals linked to hypertensive care two years after a new notification of elevated blood pressure (weighted estimates)

For peer review only

For peer review only - http://bmjopen.bmj.com/site/about/guidelines.xhtml

For peer review only

For peer review only - http://bmjopen.bmj.com/site/about/guidelines.xhtml

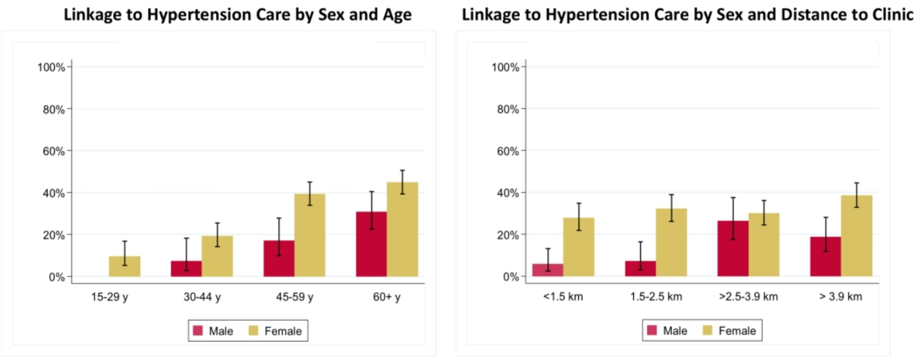

Figure 2. Proportion of individuals linked to hypertensive care two years after a new notification of elevated blood pressure (weighted estimates)

253x142mm (300 x 300 DPI)

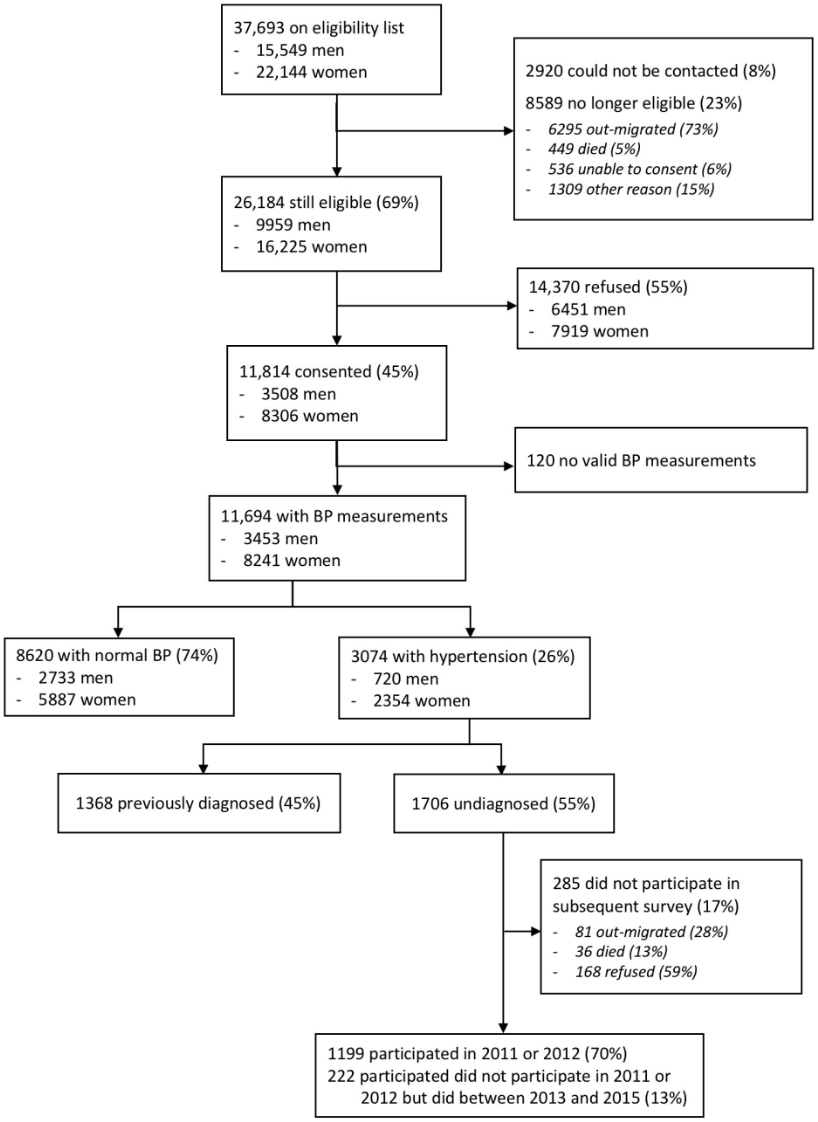

Figure 1. Flow diagram of eligible and included participants in a baseline community-based hypertension screen in 2010 and follow-up observation during 2011/2012

139x186mm (300 x 300 DPI)

Supplementary Table 1. Comparison of characteristics between those for whom a blood pressure measurement was taken in the 2010 survey, and those with no blood pressure measurements

	Eligible for survey[1]	BP measurements	No BP measurement[2]
	**N=26,184**	**N=11,694**	**N=14,490**
**Median (IQR) age (years)**	32 (20–50)	34 (21–53)	30 (20–47)
**Age group**			**P<0.001[3]**
<30	12,164 (46.5%)	5106 (43.7%)	7058 (48.7%)
35-44	5523 (21.1%)	2194 (18.8%)	3329 (23.0%)
45-59	4584 (17.5%)	2251 (19.2%)	2333 (16.1%)
60+	3913 (14.9%)	2143 (18.3%)	1770 (12.2%)
**Sex**			**P<0.001**
Male	9959 (38.0%)	3453 (29.5%)	6506 (44.9%)
Female	16,225 (62.0%)	8241 (70.5%)	7984 (55.1%)
**Marital status**			**P<0.001**
Single (never married)	8113 (31.2%)	3462 (29.8%)	4651 (32.4%)
Married/informal union	14,959 (57.6%)	6556 (56.3%)	8403 (58.6%)
Widow/sep/divorced	2910 (11.2%)	1618 (13.9%)	1292 (9.0 %)
Missing	*202*	*58*	*144*
**Education**			**P<0.001**
None	4558 (17.5%)	2389 (20.5%)	2169 (15.1%)
Less than complete secondary	13,466 (51.6%)	6244 (53.5%)	7222 (50.1%)
Complete secondary/above	8051 (30.9%)	3040 (26.0%)	5011 (34.8%)
Missing	*109*	*21*	*88*
**Employed**			**P<0.001**
Yes	5860 (22.7%)	1779 (15.3%)	4081 (28.6%)
No	19,997 (77.3%)	9828 (84.7%)	10,169 (71.4%)
Missing	*327*	*87*	*240*
**Residence**			**P<0.001**
Urban	1953 (7.5 %)	617 (5.3 %)	1336 (9.2 %)
Peri-urban	8084 (30.9%)	3604 (30.8%)	4480 (31.0%)
Rural	16,092 (61.6%)	7464 (63.9%)	8628 (59.7%)
Missing	*55*	*9*	*46*
**SES tertile**			**P<0.001**
Low	8566 (33.6%)	4193 (36.4%)	4373 (31.4%)
Middle	8330 (32.7%)	3818 (33.1%)	4512 (32.4%)
High	8569 (33.7%)	3522 (30.5%)	5047 (36.2%)
Missing	*719*	*161*	*558*

[1]Individuals who were on the eligibility list for the 2010 survey (aged ≥15 years as of Dec 2009 and resident in the DSS), were successfully contacted (92% of all on the list) and still eligible at the time of contact (75% of those contacted). [2]Includes 14,370 individuals who refused consent, and 120 individuals who consented but for whom blood pressure measurements were not available. [3]P-value from Chi-squared test comparing those with blood pressure measurements and those without.

For peer review only

Supplementary Table 2. Comparison of characteristics between those who participated in the general health survey in 2011 or 2012 and those who did not, among 1706 individuals with undiagnosed hypertension in 2010

	Participated in 2011/2012	Did not participate in 2011/2012	Participated in 2011–2016	No participation in later survey
	N=1199 (70.3%)	N=507 (29.7%)	N=1421 (83.3%)	N=285 (16.7%)
**Median (IQR) age (years)**	50 (38–66)	43 (29–58)	50 (37–65)	41 (28–57)
**Age group**		P<0.001[1]		P<0.001[2]
<30	193 (16.1%)	140 (27.6%)	248 (17.5%)	85 (29.8%)
35-44	242 (20.2%)	124 (24.5%)	284 (20.0%)	82 (28.8%)
45-59	360 (30.0%)	125 (24.7%)	430 (30.3%)	55 (19.3%)
60+	404 (33.7%)	118 (23.3%)	459 (32.3%)	63 (22.1%)
**Sex**		P<0.001		P<0.001
Male	308 (25.7%)	234 (46.2%)	394 (27.7%)	148 (51.9%)
Female	891 (74.3%)	273 (53.8%)	1027 (72.3%)	137 (48.1%)
**Marital status**		P<0.001		P<0.001
Single (never married)	250 (20.9%)	94 (18.7%)	287 (20.2%)	57 (20.2%)
Married/informal union	666 (55.5%)	342 (67.9%)	817 (57.5%)	191 (67.7%)
Widow/sep/divorced	283 (23.6%)	68 (13.5%)	317 (22.3%)	34 (12.1%)
**Education**		P<0.001		P<0.001
None	363 (30.3%)	119 (23.6%)	424 (29.8%)	58 (20.5%)
Less than complete secondary	581 (48.5%)	220 (43.6%)	678 (47.7%)	123 (43.5%)
Complete secondary/above	255 (21.3%)	166 (32.9%)	319 (22.4%)	102 (36.0%)
**Employed**		P<0.001		P<0.001
Yes	178 (14.8%)	119 (23.6%)	220 (15.5%)	77 (27.3%)
No	1021 (85.2%)	385 (76.4%)	1201 (84.5%)	205 (72.7%)
**Residence**		P=0.04		P=0.16
Urban	36 (3.0 %)	28 (5.5 %)	48 (3.4 %)	16 (5.6 %)
Peri-urban	398 (33.2%)	159 (31.4%)	470 (33.1%)	87 (30.6%)
Rural	765 (63.8%)	319 (63.0%)	903 (63.5%)	181 (63.7%)
**SES tertile**		P=0.24		P=0.05
Low	459 (38.7%)	189 (38.1%)	544 (38.7%)	104 (37.5%)
Middle	364 (30.7%)	171 (34.5%)	431 (30.7%)	104 (37.5%)
High	364 (30.7%)	136 (27.4%)	431 (30.7%)	69 (24.9%)
**BMI category**		P=0.008		P<0.001
Underweight	50 (5.7 %)	25 (7.2 %)	58 (5.6 %)	17 (9.0 %)
Normal weight	294 (33.6%)	146 (42.3%)	351 (34.0%)	89 (47.3%)
Overweight	229 (26.2%)	84 (24.3%)	269 (26.1%)	44 (23.4%)
Obese	301 (34.4%)	90 (26.1%)	353 (34.2%)	38 (20.2%)
**Distance to nearest clinic (km)**		P=0.22		P=0.50
0- <1.5	263 (21.9%)	121 (23.9%)	320 (22.5%)	64 (22.5%)
1.5-2.5	269 (22.4%)	127 (25.1%)	323 (22.7%)	73 (25.7%)
>2.5-3.9	310 (25.9%)	131 (25.9%)	365 (25.7%)	76 (26.8%)
>3.9	357 (29.8%)	127 (25.1%)	413 (29.1%)	71 (25.0%)

[1]P-value from Chi-squared test comparing individuals who participated in 2011-2012 (N=1199) with those who did not (N=507). [2]P-value from Chi-squared test comparing individuals who participated in 2011-2015 (N=1421) with those who did not (N=285).

For peer review only

Supplementary Table 3. Factors[1] associated with linkage to hypertension care within 2 years after home-based diagnosis of hypertension[2] in 2010, among individuals who were previously undiagnosed and participated in 2011 or 2012 (N=1199), using inverse probability weighting to account for non-participation in the blood pressure screen

	Linked to care/N (%)	Crude OR (95% CI)[3]	Age- & sex-adjusted OR (95% CI)[3]	Adjusted OR (95% CI)[3,4]
**Sociodemographic factors**				
**Age group**		P<0.001	P<0.001	P<0.001
<30	10 / 193 (5.2 %)	1	1	1
35-44	40 / 242 (16.5%)	3.82 (1.85 -7.87)	3.20 (1.55 -6.62)	3.26 (1.54 -6.90)
45-59	126 / 360 (35.0%)	10.35 (5.28 -20.32)	8.64 (4.39 -16.99)	9.35 (4.73 -18.46)
60+	167 / 404 (41.3%)	13.75 (7.05 -26.81)	12.27 (6.29 -23.91)	13.48 (6.82 -26.61)
**Sex**		P<0.001	P<0.001	P<0.001
Male	47 / 308 (15.3%)	1	1	1
Female	296 / 891 (33.2%)	2.86 (2.03 -4.03)	2.57 (1.81 -3.66)	2.77 (1.91 -4.00)
**Marital status**		P<0.001	P=0.09	P=0.09
Single (never married)	55 / 250 (22.0%)	1	1	1
Married/informal union	178 / 666 (26.7%)	1.35 (0.95 -1.91)	1.35 (0.91 -1.99)	1.36 (0.92 -2.01)
Widow/sep/divorced	110 / 283 (38.9%)	2.40 (1.63 -3.54)	0.94 (0.61 -1.44)	0.95 (0.61 -1.45)
**Education**		P<0.001	P=0.86	P=0.89
None	132 / 363 (36.4%)	1	1	1
Less than complete secondary	160 / 581 (27.5%)	0.64 (0.48 -0.84)	1.09 (0.80 -1.49)	1.07 (0.78 -1.46)
Complete secondary/above	51 / 255 (20.0%)	0.42 (0.29 -0.61)	1.06 (0.69 -1.63)	1.10 (0.71 -1.70)
**Employed**		P<0.001	P=0.01	P=0.01
Yes	31 / 178 (17.4%)	1	1	1
No	312 / 1021 (30.6%)	2.21 (1.46 -3.34)	1.74 (1.13 -2.69)	1.76 (1.14 -2.72)
**SES tertile**		P=0.36	P=0.32	P=0.26
Low	125 / 459 (27.2%)	1	1	1
Middle	99 / 364 (27.2%)	1.01 (0.74 -1.38)	1.11 (0.79 -1.55)	1.12 (0.81 -1.57)
High	115 / 364 (31.6%)	1.23 (0.90 -1.66)	1.28 (0.93 -1.77)	1.31 (0.95 -1.82)
**Location factors**				
**Residence**		P=0.03	P=0.40	P=0.68
Urban	10 / 36 (27.8%)	1	1	1
Peri-urban	95 / 398 (23.9%)	0.91 (0.42 -1.96)	0.84 (0.37 -1.89)	0.73 (0.32 -1.67)
Rural	238 / 765 (31.1%)	1.32 (0.62 -2.79)	1.03 (0.47 -2.27)	0.69 (0.30 -1.59)
**Nearest clinic (km)**[5]				
0- <1.5	56 / 263 (21.3%)			
1.5-2.5	71 / 269 (26.4%)	P<0.001	P=0.001	P=0.002
>2.5-3.9	93 / 310 (30.0%)	1.15 (1.08 -1.23)[6]	1.13 (1.05 -1.21)[6]	1.12 (1.04 -1.20)[6]
>3.9	123 / 357 (34.5%)			
**Clinical factors**				
**BMI category**		P<0.001	P=0.06	P=0.14
<25 kg/m^2	70 / 344 (20.3%)	1	1	1
25 – <30 kg/m^2	62 / 229 (27.1%)	1.56 (1.05 -2.33)	1.13 (0.73 -1.74)	1.19 (0.77 -1.84)
≥30 kg/m^2	110 / 301 (36.5%)	2.42 (1.69 -3.45)	1.57 (1.05 -2.34)	1.51 (1.00 -2.28)
**Hypertension stage**[7]		P<0.001	P<0.001	P<0.001
Stage I	142 / 730 (19.5%)	1	1	1
Stage II	134 / 342 (39.2%)	2.72 (2.04 -3.63)	2.20 (1.62 -2.99)	2.15 (1.58 -2.93)
Hypertension urgency	67 / 127 (52.8%)	4.79 (3.22 -7.13)	3.14 (2.06 -4.78)	3.10 (2.04 -4.71)
**Self-report of diabetes**[8]		P=0.28	P=0.50	P=0.56
No	339 / 1191 (28.5%)	1	1	1
Yes	4 / 8 (50.0%)	2.16 (0.53 -8.83)	1.56 (0.43 -5.65)	1.48 (0.40 -5.52)

For peer review only

**Self-report of TB[8]**		P=0.81	P=0.77	P=0.48
No	332 / 1156 (28.7%)	1	1	1
Yes	11 / 43 (25.6%)	0.92 (0.45 -1.86)	1.12 (0.51 -2.49)	1.34 (0.59 -3.04)

[1]All characteristics are based on 2010 survey. [2]Hypertension defined as systolic BP ≥ 140mmHg or diastolic BP ≥90 mmHg, in an average of 2 readings. [3]Weighted for non-response; weights calculated as the inverse probability of survey participation, in strata defined by age group, sex, education level and place of residence. [4]Sociodemographic factors adjusted for age group, sex, marital status and employment. Location factors adjusted for age group, sex, marital status, employment, and distance from nearest clinic as continuous covariate. Clinical factors adjusted for age group, sex, marital status, employment, distance from nearest clinic, and hypertension stage. [5]Quartiles based on distribution in all individuals who were eligible for 2010 survey. Fit as continuous covariate; n (%) linked in each distance quartile shown for information only. [6]OR for linear trend in linkage with each 1 km increase in distance. [7]Stage I: Systolic BP 140–159 or diastolic BP 90–99; Stage II: Systolic BP 160–179 or diastolic BP 100–119; Hypertension urgency: Systolic BP ≥180 or diastolic BP ≥120. [8]Reports being diagnosed in the past 12m or currently on treatment.

For peer review only

Supplementary Table 4. Factors[1] associated with linkage to hypertension care within 5 years (2011 to 2015) after home-based diagnosis of hypertension[2] in 2010, among individuals who were previously undiagnosed and participated in a subsequent survey (N=1421) using inverse probability weighting to account for non-participation in the blood pressure screen

	Linked to care/N (%)	Crude OR (95% CI)[3]	Age- & sex-adjusted OR (95% CI)[3]	Adjusted OR (95% CI)[3,4]
**Sociodemographic factors**				
**Age group**		P<0.001	P<0.001	P<0.001
<30	25 / 248 (10.1%)	1	1	1
35-44	75 / 284 (26.4%)	3.22 (1.97 -5.29)	2.69 (1.64 -4.43)	2.93 (1.77 -4.87)
45-59	211 / 430 (49.1%)	8.67 (5.48 -13.70)	7.50 (4.73 -11.89)	8.05 (5.05 -12.84)
60+	265 / 459 (57.7%)	12.37 (7.84 -19.53)	11.65 (7.35 -18.47)	11.49 (7.25 -18.22)
**Sex**		P<0.001	P<0.001	P<0.001
Male	92 / 394 (23.4%)	1	1	1
Female	484 / 1027 (47.1%)	3.06 (2.35 -3.99)	2.98 (2.25 -3.94)	2.85 (2.15 -3.79)
**Marital status**		P<0.001	P=0.66	P=0.64
Single (never married)	98 / 287 (34.1%)	1	1	1
Married/informal union	298 / 817 (36.5%)	1.16 (0.88 -1.55)	1.04 (0.76 -1.44)	1.07 (0.77 -1.48)
Widow/sep/divorced	180 / 317 (56.8%)	2.78 (1.99 -3.87)	0.90 (0.61 -1.32)	0.92 (0.62 -1.35)
**Education**		P<0.001	P=0.55	P=0.66
None	217 / 424 (51.2%)	1	1	1
Less than complete secondary	269 / 678 (39.7%)	0.60 (0.47 -0.76)	1.13 (0.85 -1.50)	1.13 (0.86 -1.50)
Complete secondary/above	90 / 319 (28.2%)	0.36 (0.26 -0.49)	0.98 (0.68 -1.41)	1.05 (0.73 -1.53)
**Employed**		P<0.001	P=0.007	P=0.007
Yes	61 / 220 (27.7%)	1	1	1
No	515 / 1201 (42.9%)	2.04 (1.48 -2.81)	1.62 (1.14 -2.29)	1.62 (1.14 -2.29)
**SES tertile**		P=0.49	P=0.71	P=0.58
Low	224 / 544 (41.2%)	1	1	1
Middle	163 / 431 (37.8%)	0.89 (0.68 -1.15)	0.95 (0.71 -1.27)	0.97 (0.73 -1.30)
High	183 / 431 (42.5%)	1.04 (0.80 -1.35)	1.08 (0.81 -1.43)	1.13 (0.85 -1.50)
**Location factors**				
**Residence**		P=0.07	P=0.89	P=0.61
Urban	18 / 48 (37.5%)	1	1	1
Peri-urban	173 / 470 (36.8%)	1.03 (0.56 -1.92)	0.95 (0.50 -1.79)	0.84 (0.44 -1.62)
Rural	385 / 903 (42.6%)	1.34 (0.73 -2.46)	1.01 (0.54 -1.88)	0.75 (0.39 -1.46)
**Nearest clinic (km)** [5]				
0- <1.5	111 / 320 (34.7%)			
1.5-2.5	128 / 323 (39.6%)	P=0.001	P=0.03	P=0.05
>2.5-3.9	147 / 365 (40.3%)	1.10 (1.04 -1.17)[6]	1.08 (1.01 -1.15)[6]	1.07 (1.00 -1.14)[6]
>3.9	190 / 413 (46.0%)			
**BMI category**		P<0.001	P=0.004	P=0.008
<25 kg/m^2	133 / 409 (32.5%)	1	1	1
25 – <30 kg/m^2	99 / 269 (36.8%)	1.28 (0.92 -1.77)	0.88 (0.61 -1.26)	0.90 (0.62 -1.31)
≥30 kg/m^2	186 / 353 (52.7%)	2.44 (1.81 -3.29)	1.55 (1.10 -2.18)	1.53 (1.08 -2.17)
**Hypertension stage**[7]		P<0.001	P<0.001	P<0.001
Stage I	264 / 863 (30.6%)	1	1	1
Stage II	215 / 415 (51.8%)	2.49 (1.95 -3.17)	2.01 (1.54 -2.62)	2.12 (1.55 -2.89)
Hypertension urgency	97 / 143 (67.8%)	5.02 (3.42 -7.37)	3.18 (2.10 -4.82)	3.29 (2.01 -5.39)
**Self-report of diabetes**[8]		P=0.05	P=0.08	P=0.32
No	568 / 1410 (40.3%)	1	1	1
Yes	8 / 11 (72.7%)	3.84 (0.98 -15.03)	3.09 (0.88 -10.81)	5.18 (0.21 -128.15)

For peer review only

For peer review only - http://bmjopen.bmj.com/site/about/guidelines.xhtml

**Self-report of TB[8]**		P=0.73	P=0.86	P=0.30
No	560 / 1376 (40.7%)	1	1	1
Yes	16 / 45 (35.6%)	0.89 (0.48 -1.68)	1.07 (0.49 -2.33)	1.57 (0.67 -3.71)

[1]All characteristics are based on 2010 survey. [2]Hypertension defined as systolic BP ≥ 140mmHg or diastolic BP ≥90 mmHg, in an average of 2 readings. [3]Weighted for non-response; weights calculated as the inverse probability of survey participation, in strata defined by age group, sex, education level and place of residence.
[4]Sociodemographic factors adjusted for age group, sex, and employment. Location factors adjusted for age group, sex, employment, and distance from nearest clinic as continuous covariate. Clinical factors adjusted for age group, sex, employment, distance from nearest clinic, hypertension stage, and BMI. [5]Quartiles based on distribution in all individuals who were eligible for 2010 survey. Fit as continuous covariate; n (%) linked in each distance quartile shown for information only. [6]OR for linear trend in linkage with each 1 km increase in distance. [7]Stage I: Systolic BP 140–159 or diastolic BP 90–99; Stage II: Systolic BP 160–179 or diastolic BP 100–119; Hypertension urgency: Systolic BP ≥180 or diastolic BP ≥120. [8]Reports being diagnosed in the past 12m or currently on treatment.

For peer review only

Supplementary Table 5. Factors[1] associated with linkage to hypertension care within 5 years (2011 to 2015) after home-based diagnosis of hypertension[2] in 2010, among individuals who were previously undiagnosed and participated in a subsequent survey (N=1421)

	Linked to care/N (%)	Crude OR (95% CI)[3]	Adjusted OR (95% CI)[3,4]	Adjusted OR (95% CI)[3,5]
**Sociodemographic factors**				
**Age group**		P<0.001	P<0.001	P<0.001
<30	25 / 248 (10.1%)	1	1	1
35-44	75 / 284 (26.4%)	3.22 (1.97 -5.29)	2.43 (1.47 -4.01)	2.61 (1.56 -4.34)
45-59	211 / 430 (49.1%)	8.67 (5.48 -13.70)	6.58 (4.13 -10.47)	6.96 (4.35 -11.14)
60+	265 / 459 (57.7%)	12.37 (7.84 -19.53)	10.23 (6.44 -16.25)	10.11 (6.37 -16.06)
**Sex**		P<0.001	P<0.001	P<0.001
Male	92 / 394 (23.4%)	1	1	1
Female	484 / 1027 (47.1%)	3.06 (2.35 -3.99)	2.54 (1.91 -3.39)	2.46 (1.84 -3.30)
**Times participated**				
Once	61 / 321 (19.0%)			
Twice	119 / 335 (35.5%)	P<0.001	P<0.001	P<0.001
3 times	160 / 327 (48.9%)	1.57 (1.44 -1.71)[6]	1.40 (1.27 -1.53)[6]	1.39 (1.26 -1.52)[6]
4 times	147 / 287 (51.2%)			
5 times	89 / 151 (58.9%)			
**Marital status**		P<0.001	P=0.60	P=0.55
Single (never married)	98 / 287 (34.1%)	1	1	1
Married/informal union	298 / 817 (36.5%)	1.16 (0.88 -1.55)	1.14 (0.82 -1.60)	1.16 (0.83 -1.63)
Widow/sep/divorced	180 / 317 (56.8%)	2.78 (1.99 -3.87)	1.00 (0.67 -1.48)	1.01 (0.68 -1.50)
**Education**		P<0.001	P=0.74	P=0.67
None	217 / 424 (51.2%)	1	1	1
Less than complete secondary	269 / 678 (39.7%)	0.60 (0.47 -0.76)	1.12 (0.84 -1.49)	1.12 (0.84 -1.50)
Complete secondary/above	90 / 319 (28.2%)	0.36 (0.26 -0.49)	1.09 (0.75 -1.59)	1.16 (0.79 -1.69)
**Employed**		P<0.001	P=0.03	P=0.03
Yes	61 / 220 (27.7%)	1	1	1
No	515 / 1201 (42.9%)	2.04 (1.48 -2.81)	1.48 (1.04 -2.10)	1.48 (1.04 -2.10)
**SES tertile**		P=0.49	P=0.68	P=0.56
Low	224 / 544 (41.2%)	1	1	1
Middle	163 / 431 (37.8%)	0.89 (0.68 -1.15)	0.98 (0.73 -1.32)	1.00 (0.74 -1.35)
High	183 / 431 (42.5%)	1.04 (0.80 -1.35)	1.11 (0.83 -1.48)	1.15 (0.86 -1.54)
**Location factors**				
**Residence**		P=0.07	P=0.63	P=0.61
Urban	18 / 48 (37.5%)	1	1	1
Peri-urban	173 / 470 (36.8%)	1.03 (0.56 -1.92)	0.82 (0.43 -1.57)	0.76 (0.40 -1.48)
Rural	385 / 903 (42.6%)	1.34 (0.73 -2.46)	0.93 (0.49 -1.74)	0.85 (0.45 -1.61)
**Nearest clinic (km)[7]**				
0- <1.5	111 / 320 (34.7%)			
1.5-2.5	128 / 323 (39.6%)	P=0.001	P=0.06	P=0.10
>2.5-3.9	147 / 365 (40.3%)	1.10 (1.04 -1.17)[8]	1.07 (1.00 -1.14)[8]	1.06 (0.99 -1.13)[8]
>3.9	190 / 413 (46.0%)			
**BMI category**		P<0.001	P=0.003	P=0.007
<25 kg/m^2	133 / 409 (32.5%)	1	1	1
25 – <30 kg/m^2	99 / 269 (36.8%)	1.28 (0.92 -1.77)	0.88 (0.61 -1.28)	0.89 (0.61 -1.30)
≥30 kg/m^2	186 / 353 (52.7%)	2.44 (1.81 -3.29)	1.58 (1.11 -2.25)	1.54 (1.07 -2.22)
**Hypertension stage[9]**		P<0.001	P<0.001	P<0.001
Stage I	264 / 863 (30.6%)	1	1	1
Stage II	215 / 415 (51.8%)	2.49 (1.95 -3.17)	2.19 (1.67 -2.88)	2.36 (1.71 -3.26)
Hypertension urgency	97 / 143 (67.8%)	5.02 (3.42 -7.37)	3.44 (2.25 -5.25)	3.74 (2.25 -6.20)

For peer review only

**Self-report of diabetes[10]**		P=0.05	P=0.03	P=0.28
No	568 / 1410 (40.3%)	1	1	1
Yes	8 / 11 (72.7%)	3.84 (0.98 -15.03)	3.67 (1.14 -11.80)	5.64 (0.25 -126.50)
**Self-report of TB[10]**		P=0.73	P=0.91	P=0.56
No	560 / 1376 (40.7%)	1	1	1
Yes	16 / 45 (35.6%)	0.89 (0.48 -1.68)	0.96 (0.43 -2.13)	1.31 (0.52 -3.27)

[1]All characteristics are based on 2010 survey. [2]Hypertension defined as systolic BP ≥ 140mmHg or diastolic BP ≥90 mmHg, in an average of 2 readings. [3]Weighted for non-response; weights calculated as the inverse probability of survey participation, in strata defined by age group, sex, education level and place of residence. [4]Adjusted for age, sex and number of times participated in subsequent surveys as a continuous covariate. [5]Sociodemographic factors adjusted for age group, sex, number of times in subsequent surveys, and employment. Location factors adjusted for age group, sex, number of times in subsequent surveys, and employment. Clinical factors adjusted for age group, sex, number of times in subsequent surveys, employment, hypertension stage, and BMI. [6]OR for linear trend in reported linkage with each unit increase in survey participation. [7]Quartiles based on distribution in all individuals who were eligible for 2010 survey. Fit as continuous covariate; n (%) linked in each distance quartile shown for information only. [8]OR for linear trend in linkage with each 1 km increase in distance. [9]Stage I: Systolic BP 140–159 or diastolic BP 90–99; Stage II: Systolic BP 160–179 or diastolic BP 100–119; Hypertension urgency: Systolic BP ≥180 or diastolic BP ≥120. [10]Reports being diagnosed in the past 12m or currently on treatment.

For peer review only

For peer review only - http://bmjopen.bmj.com/site/about/guidelines.xhtml

STROBE Statement—Checklist of items that should be included in reports of ***cohort studies***

	Item No	Recommendation	Page	Comments
**Title and abstract**	1	(*a*) Indicate the study's design with a commonly used term in the title or the abstract	1	
		(*b*) Provide in the abstract an informative and balanced summary of what was done and what was found	2	
**Introduction**				
Background/rationale	2	Explain the scientific background and rationale for the investigation being reported	4	Lines 2-23
Objectives	3	State specific objectives, including any prespecified hypotheses	4-5	Lines 25-32
**Methods**				
Study design	4	Present key elements of study design early in the paper	5	Lines 36-43
Setting	5	Describe the setting, locations, and relevant dates, including periods of recruitment, exposure, follow-up, and data collection	5	Lines 36-43
Participants	6	(*a*) Give the eligibility criteria, and the sources and methods of selection of participants. Describe methods of follow-up	6	Lines 70-79
		(*b*) For matched studies, give matching criteria and number of exposed and unexposed	N/A	
Variables	7	Clearly define all outcomes, exposures, predictors, potential confounders, and effect modifiers. Give diagnostic criteria, if applicable	5-6	Lines 45-67
Data sources/ measurement	8*	For each variable of interest, give sources of data and details of methods of assessment (measurement). Describe comparability of assessment methods if there is more than one group	5-6	Lines 45-67
Bias	9	Describe any efforts to address potential sources of bias	7	Lines 94 - 103
Study size	10	Explain how the study size was arrived at	9	Lines 129 - 141
Quantitative variables	11	Explain how quantitative variables were handled in the analyses. If applicable, describe which groupings were chosen and why	6	Lines 69 - 79
Statistical methods	12	(*a*) Describe all statistical methods, including those used to control for confounding	6-7	Lines 69 - 103
		(*b*) Describe any methods used to examine subgroups and interactions	6	Lines 73 – 76
		(*c*) Explain how missing data were addressed	7	Lines 94 - 103
		(*d*) If applicable, explain how loss to follow-up was addressed	N/A	
		(*e*) Describe any sensitivity analyses	7	Lines 94 - 103
**Results**				
Participants	13*	(a) Report numbers of individuals at each stage	8-9	Lines 113 - 141

For peer review only - http://bmjopen.bmj.com/site/about/guidelines.xhtml

		of study—eg numbers potentially eligible, examined for eligibility, confirmed eligible, included in the study, completing follow-up, and analysed		
		(b) Give reasons for non-participation at each stage	8-9	Lines 113 - 141
		(c) Consider use of a flow diagram		Figure 1
Descriptive data	14*	(a) Give characteristics of study participants (eg demographic, clinical, social) and information on exposures and potential confounders	9	Lines 139 - 149
		(b) Indicate number of participants with missing data for each variable of interest	N/A	No data was missing for participants who completed surveys
		(c) Summarise follow-up time (eg, average and total amount)	9	Lines 138 - 149
Outcome data	15*	Report numbers of outcome events or summary measures over time	9-10	Lines 151 - 161
Main results	16	(*a*) Give unadjusted estimates and, if applicable, confounder-adjusted estimates and their precision (eg, 95% confidence interval). Make clear which confounders were adjusted for and why they were included	10	Lines 163 - 180
		(*b*) Report category boundaries when continuous variables were categorized		Table 2
		(*c*) If relevant, consider translating estimates of relative risk into absolute risk for a meaningful time period	9-10	Lines 151 – 161 (we report absolute prevalence of linkage prior to our multivariable model estimations)
Other analyses	17	Report other analyses done—eg analyses of subgroups and interactions, and sensitivity analyses	10	Lines 177 – 180, Supplementary Tables
**Discussion**				
Key results	18	Summarise key results with reference to study objectives	11	Lines 183-191
Limitations	19	Discuss limitations of the study, taking into account sources of potential bias or imprecision. Discuss both direction and magnitude of any potential bias	13	Lines 240-248
Interpretation	20	Give a cautious overall interpretation of results considering objectives, limitations, multiplicity of analyses, results from similar studies, and other relevant evidence	13	Lines 150-254
Generalisability	21	Discuss the generalisability (external validity) of the study results	13	Lines 248-249
**Other information**				
Funding	22	Give the source of funding and the role of the funders for the present study and, if applicable, for the original study on which the present	1	Funding statement

For peer review only - http://bmjopen.bmj.com/site/about/guidelines.xhtml

	article is based

*Give information separately for exposed and unexposed groups.

Note: An Explanation and Elaboration article discusses each checklist item and gives methodological background and published examples of transparent reporting. The STROBE checklist is best used in conjunction with this article (freely available on the Web sites of PLoS Medicine at http://www.plosmedicine.org/, Annals of Internal Medicine at http://www.annals.org/, and Epidemiology at http://www.epidem.com/). Information on the STROBE Initiative is available at http://www.strobe-statement.org.

For peer review only

For peer review only - http://bmjopen.bmj.com/site/about/guidelines.xhtml
